# Structural Properties of Prokaryotic Promoter Regions Correlate with Functional Features

**DOI:** 10.1371/journal.pone.0088717

**Published:** 2014-02-07

**Authors:** Pieter Meysman, Julio Collado-Vides, Enrique Morett, Roberto Viola, Kristof Engelen, Kris Laukens

**Affiliations:** 1 Department of Mathematics and Computer Science, University of Antwerp, Antwerp, Belgium; 2 Biomedical Informatics Research Center Antwerp (biomina), University of Antwerp/Antwerp University Hospital, Edegem, Belgium; 3 Centro de Ciencias Genómicas, Universidad Nacional Autónoma de México, Cuernavaca, Morelos, Mexico; 4 Instituto de Biotecnología, Universidad Nacional Autónoma de México, Cuernavaca, Morelos, Mexico; 5 Instituto Nacional de Medicina Genómica, Mexico City, Mexico; 6 Department of Computational Biology, Fondazione Edmund Mach, San Michele all’Adige, Trento, Italy; Indian Institute of Science, India

## Abstract

The structural properties of the DNA molecule are known to play a critical role in transcription. In this paper, the structural profiles of promoter regions were studied within the context of their diversity and their function for eleven prokaryotic species; *Escherichia coli*, *Klebsiella pneumoniae*, *Salmonella* Typhimurium, *Pseudomonas auroginosa, Geobacter sulfurreducens Helicobacter pylori*, *Chlamydophila pneumoniae*, *Synechocystis sp., Synechoccocus elongates, Bacillus anthracis*, and the archaea *Sulfolobus solfataricus*. The main anchor point for these promoter regions were transcription start sites identified through high-throughput experiments or collected within large curated databases. Prokaryotic promoter regions were found to be less stable and less flexible than the genomic mean across all studied species. However, direct comparison between species revealed differences in their structural profiles that can not solely be explained by the difference in genomic GC content. In addition, comparison with functional data revealed that there are patterns in the promoter structural profiles that can be linked to specific functional loci, such as sigma factor regulation or transcription factor binding. Interestingly, a novel structural element clearly visible near the transcription start site was found in genes associated with essential cellular functions and growth in several species. Our analyses reveals the great diversity in promoter structural profiles both between and within prokaryotic species. We observed relationships between structural diversity and functional features that are interesting prospects for further research to yet uncharacterized functional loci defined by DNA structural properties.

## Introduction

The DNA molecule is not a uniform linear macromolecule as it is often represented but displays local structural variations that depend on its base composition and sequence [1], [2]. This intrinsic variability in the DNA structure plays a functional role in a variety of biological processes [3]. The structure of a DNA molecule is primarily determined by its nucleotide sequence, thus similar DNA sequences generally have similar DNA structures. The reverse is however not necessarily true: DNA molecules with similar structural properties can arise from different sequences. This redundancy is the reason that the DNA structure is often considered as a separate information level from the DNA sequence, despite the inherent relationship between the two. Various properties of the molecular structure can be modeled using structural scales derived from theoretical simulations and/or experimental measurements [4]–[6]. The DNA molecule is highly variable at many different levels and thus many possible DNA structural properties can be characterized, from the local stability of the helical duplex to the global conformation of the molecule. These structural characteristics of genomic regions can be represented as structural profiles, where each position in the region is a assigned a value that denotes a specific structural property of the DNA at this location [3].

Several previous studies have analyzed structural profiles of prokaryotic promoter regions [7], [8]. On average, most prokaryotic promoters appeared to be less stable, more rigid, and more extremely curved than other genomic regions [7], [9]–[13]. However these studies were based on the limited number of transcription start sites (TSS) that were available at the time. Recent years have seen a growing interest in the DNA structure, as its influence on a variety of genomic elements has been described; e.g. transcription factor binding sites (TFBS), nucleosome positioning and transposon insertion sites [3], [14]–[16]. Also in the case of promoter regions, structural properties are widely used as the main, if not the only, feature in promoter classification [17]–[22]. For example, one of the most common approaches in this regard is the discovery of prokaryotic promoter regions based on the difference in the DNA duplex stability upstream and downstream from the TSS [9], [23], [24].

Recent technical advances have inspired us to re-evaluate the structural properties of the prokaryotic promoter region. Next generation sequencing allows the characterization of TSSs on a genome-wide scale with single-nucleotide precision with much greater ease than before. Additionally, new techniques now allow isolation of primary transcripts from the RNA pool to detect *bona fide* TSSs. Primary transcripts have a 5′ tri-phosphate group, but they usually get quickly processed. This results in a new 5′ that might not represent the actual TSS containing a mono-phosphate instead of a triphosphate. Enrichment for 5′ tri-phosphate transcripts, e.g. by selective digestion of RNA molecules capped with a 5′ mono-phosphate group or by ligating a biotinilated oligonuclotide, helps to enrich for primary mRNA and sRNA transcripts, whereas the processed transcripts or most of the cellular rRNA (which due to its polycistronic nature is mostly mature 5′ monophosphate) are removed [25], [26]. The use of these technologies has facilitated the study of TSSs such that there is now a wealth of detailed TSS data available for a variety of prokaryotic organisms. Furthermore, large-scale homogenized expression compendia were recently made available for several prokaryotic species. These compendia represent a rich resource to explore the coordination of genome-wide gene expression responses across a great variety of conditions [27]. For instance, studies have revealed insightful new patterns in prokaryotic expression regulation, such as the existence of large expression classes in prokaryotic genes which underlie massive life style switches of single cell organisms [28].

In this paper the structural properties of prokaryotic promoter regions were characterized with three goals in mind. The first goal was to describe the structural profiles of TSSs in model prokaryotic organisms, and to compare them to past observations made with more limited data sets in order to verify whether the common assumptions regarding their characteristics remain valid. The second goal was to gain novel biological insights regarding the role that the structural properties of the promoter DNA play in transcription. For example, we investigated whether certain patterns in the structural profiles can be linked to gene expression characteristics associated with these promoters. The third goal was to expand the analyses of promoter region to an evolutionary context by comparing the structural profiles of different related and more distant species.

## Results

### The Promoter Regions Reported by Different Experimental Methods Display Similar Structural Profiles

As mentioned in the introduction, there are many experimental methods to determine the location of TSSs in a genome. An exact determination of the *bona fide* TSSs is crucial to all subsequent analyses, since TSSs limit the downstream region of the promoter. The wealth of experimental data, particularly for *E. coli,* generated with different methods also allowed us to investigate whether the experimental approaches used to determine the TSS have any influence on the structural properties of the promotor region.

We can divide the *E. coli* TSS data into three broad categories based on their experimental origin: low-throughput experiments (LT) from classic single-object experiments, collected in curated databases; high-throughput experiments (HT), e.g. RNAseq without preprocessing; and high-throughput experiments enriched for primary transcripts (HTE), e.g. RNAseq preceded by terminator exonuclease treatment or adapter ligation. The overlap between the TSSs of these data sources, allowing only for a deviation of three nucleotides (plus minus three), is presented in table 1. The low overlap between the three different experimental categories can be partially explained by the fact that no data set has TSS for every transcription unit present in the *E. coli* genome. The regions from 200 nt upstream to 50 nt downstream from the TSSs were considered as the ‘promoter region’. The downstream 50 nt are cautiously included, as some regulatory elements, such as repressor binding sites, are known to occur after the TSS [29]. The selection of structural properties for this study was based on their association with prokaryotic promoter activity reported in the literature. The used structural scales are base stacking energy, denaturation temperature, curvature, B-DNA twist, Z-DNA-philicity and major groove bendability [30]–[35].

**Table 1:** TSS Overlap between experimental methods.

	#TSS	LT	HT	HTE
Low-throughput	972	100.00%	44.63%	35.03%
High-throughput	1947	20.55%	100.00%	34.94%
High-throughput with enrichment	1682	18.54%	40.18%	100.00%

Reported is the number of TSSs in each set and the percentage of TSS in the row set within three nucleotides from a TSS (plus minus three) in the column set.

**Figure 1:**
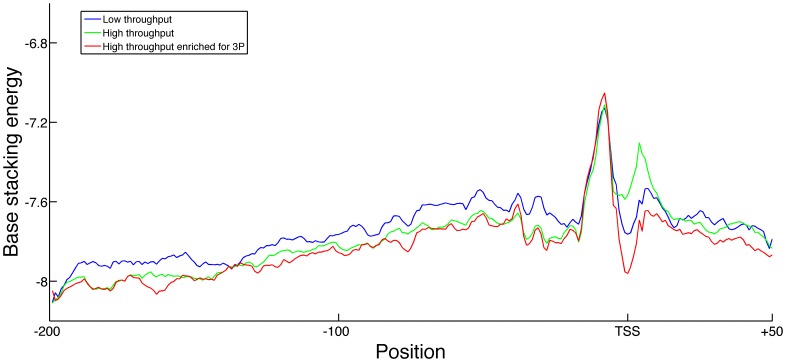
Average promoter stability profiles grouped by experimental source. Average base stacking energy profiles of TSSs from different experimental sources: low-throughput (LT), high-throughput without preprocessing (HT) and high-throughput with 3P enrichment (HTE). Reported structural profile spans from 200 nt upstream to 50 nt downstream of the TSS. Higher base stacking energies correspond to lower DNA stability. Thus the structural profile derived from the HT experiments reflects lower stability at the TSS when compared to those from the LT and HTE experiments.

Comparison of the structural properties of the promoter regions between the three methodological categories reveals that most structural profiles are unaffected by the experimental method. The majority of the variation between the different average profiles of the promoter region was not significant and fell well within the intrinsic variation present between different promoter sequences from the same method. The only striking exception is the region about 10 nucleotides downstream the TSS, where the average base stacking energy from HT experiments is higher than both the LT and HTE profiles, as can be seen in figure 1. This difference in base stacking energy within a region of 10 nt, was significant both between the HT and LT (KS-test p-value 7.7⋅10^−9^) and between HT and HTE (KS-test p-value 8.6⋅10^−28^). Given the relation between DNA sequence and structure, it seems likely that there is also a difference in the nucleotide sequence at this position. Indeed comparison of the nucleotide sequences reveals that the HTE-derived TSSs have a much higher frequency of either guanine or cytosine at the TSS position or downstream, compared to either the HT- or LT-generated TSS (see Figure S1). As the remainder of the structural profiles derived form the other scales (and the consensus sequence) do display high similarity, the experimental method will likely have little impact on the overall findings. These small differences between the methods are thus not the major topic of the subsequent sections, and we combine the TSS data from different experimental methods in the following analyses.

### The Structural Profiles of the E. coli Promoter Regions Differ Greatly from the Genomic Mean

**Figure 2:**
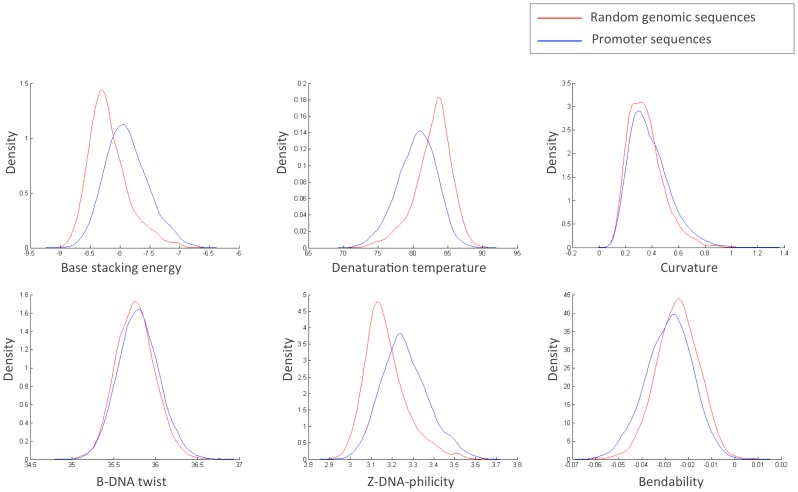
Comparison of promoter and genomic structural values. Distribution of the mean of the structural DNA properties of the E. coli promoter regions (blue line) compared to randomly selected *E. coli* genomic sequences of similar length (red line) plotted as kernel-smoothed density plots. The y-axis is thus the density, i.e. a normalized representation for the amount of promoters or random sequences with a given mean structural value as given by the x-axis. From left to down: base stacking energy in kcal/mol (KS-test p-value: 8⋅10^−189^), denaturation temperature in °C (KS-test p-value: 7⋅10^−167^), curvature angle in degrees (KS-test p-value: 3.5⋅10^−13^), B-DNA twisting angle in degrees (KS-test p-value: 4⋅10^−7^), Z-DNA-philicity in kcal/mol (KS-test p-value: 4⋅10^−187^) and bendability as log-frequency (KS-test p-value: 7⋅10^−29^).

The *E. coli* TSSs constitute the best characterized and comprehensive collection of data available. Furthermore, since *E. coli* is the most intensively studied prokaryote, there is also a rich amount of knowledge on functional elements around these promoter regions as well as on the downstream genes. For these reasons, *E. coli* constitutes a good starting point in this analysis. Therefore, TSSs from the seven data sets introduced previously, produced by many different types of experimental procedures, were combined into a single set of high quality TSSs. The *E. coli* promoter sequences could then be compared to their genomic background, based on randomly selected genomic regions of equal length. It is already apparent from the sequences themselves that the GC content of the promoter regions (45.31%) is lower than that of the genomic mean (50.79%) in *E. coli*. To discover if the structural properties for promoter regions differ from randomly selected genomic sequences, the distribution of the mean value for the different structural features was compared between them, as shown in figure 2. For every tested structural property, the TSS sequences were found to be significantly different. In general, the TSS sequences were found to be less stable, as evidenced by the lower denaturation temperature and stacking energy (which has a negative scale). This lower DNA helical stability has already been noted in previous studies and is postulated to be linked to the DNA denaturation step during RNA polymerase open complex formation [24]. Further, we also found the TSS sequences to be more rigid as per the lower bendability and the higher B-DNA twisting (which has been used as a measure for the rigidity of the DNA molecule in the past). Again, this corresponds well with previous reports [7]. Additionally, the TSS regions displayed a larger fraction of regions with extreme curvature and a lower tendency to be in the Z-DNA conformation than the remainder of the genome.

**Figure 3:**
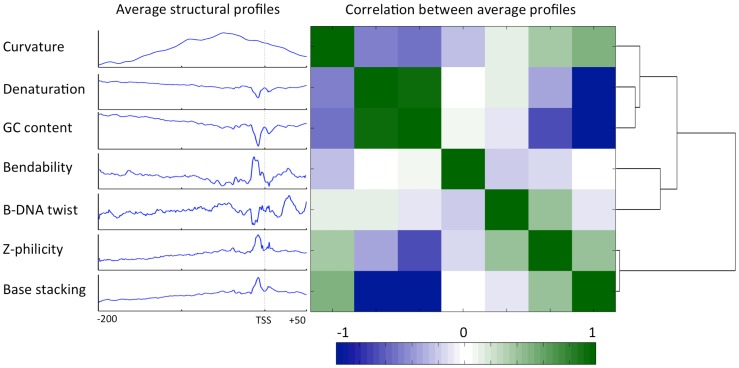
Correlation between average *E. coli* promoter structural profiles. Average structural profiles of the E. coli promoter sequences. A loess-smoothing with range 10 nt was applied prior to the calculation of the average profile. From top to bottom: DNA curvature, denaturation temperature, GC content of the sequence, bendability, B-DNA twisting, Z-DNA-philicity, and base stacking energy. Promoter sequences illustrated are 200 nt upstream from the TSS to 50 nt downstream with the TSS location marked as a grey dashed line. To the right is the heatmap showing the correlation between the average profiles at the left side of this this figure. Colors in the heatmap correspond to the correlation values with 1 (green) signifying strongly correlated average profiles; −1 (blue) signifying strong anti-correlation and 0 (white) signifying no apparent correlation. The distance based on the correlation values between the average profiles is represented by a tree to the right.

It has been reported in prior studies that there are local differences in the structural property profiles across the promoter region [8], [36]. The average structural variation of the DNA molecule at each position of the selected promoter regions of *E. coli* is shown on the left side of figure 3. For all structural properties except curvature, the most extreme values occur around the −10 position. This is very relevant, as this is the main recognition site for the RNA polymerase holoenzyme and features a relatively strong TATAAT consensus sequence that can be found in many of the promoter regions (see Figure S1). The −10 region is less stable than the surrounding region, since this is the limit of the melting of the DNA in the process of open complex formation, and it is more flexible, as seen in the B-DNA twist profile and the bendability profile. These characteristics do correspond to the expected properties of the TATAAT consensus sequence. The TSS itself is also unstable but has a high rigidity. The area upstream from the −10 position where the −35 promoter element is located, is more stable but is a region of disagreement between the two rigidity scales. From the bendability results, this region seems to be rigid, but the B-DNA twisting suggests a more flexible DNA structure. This can be the result of the directionality constraint on the bendability scale, as it only describes bendability towards the major groove. The position may therefore be flexible but not in the direction of the major groove. The curvature seems to be most pronounced between 50 nt to 100 nt upstream from the promoter region.

However, the average profiles are not representative for all promoters, since only 50% do not show any curvature (see Figure S2). This diversity in structural profiles causes a large standard deviation on all average structural values presented and underlines the need for stringent statistics in all of our analyses. Furthermore, many of the structural profiles seem to display extremes at similar positions, such as the −10 position, which suggests a dependency between the different profiles. The correlation between the average structural profiles found for the promoter regions is also presented in figure 3. While the actual values within the scales are known to be independent, by applying them to biologic sequences and smoothing them, certain patterns of correlation emerge. This increase in correlation between structural scales when applied to genomic sequences has previously been noted by Baldi *et al.* [37]. The two scales used to measure the stability of the DNA helix, namely the denaturation temperature and the base stacking are strongly anti-correlated. In addition, there is a strong correlation between the denaturation temperature and the GC content of the sequence. This relation could be expected, as the GC content is known to be a primary determinant of the stability of the DNA helix due to the difference in hydrogen bonds between AT and GC pairs. These different stability scales will therefore likely give very similar results due to the high correlation (and anti-correlation) between their structural profiles (e.g. denaturation, base staking energy). Thus we only considered the most commonly used of these scales as a measure for stability, namely base stacking energy, and will proceed with only the curvature, bendability and base stacking profiles in the remainder of the paper.

### Patterns in the Structural Profiles of the Promoter Region are Related to Functional Loci

It has been established that not all promoter regions follow the same structural profile but that they can be divided or grouped into several categories. In eukaryotic species, different promoter categories have been linked to distinct RNA polymerases [8]. Prokaryotic species only have a single RNA polymerase to transcribe their genes, but have multiple sigma factors (e.g. *E. coli* has 7), which are recruited as part of the RNA polymerase complex and are responsible for directing the binding of the complex to the promoter. Different sigma factors have a preference to bind distinct sets of promoters. The constitution of the cellular sigma factor pool thus regulates the affinity of the RNA polymerase to different promoters on a genome-wide scale [38].

Following the analyses of the structural profiles, we can evaluate whether certain sigma factors have a tendency to co-occur with specific structural patterns in the promoter. Such patterns may thus be defining characteristics involved in the specific recognition by different sigma factors. For the analysis of the effect of the sigma factors on the promoter region, we opted to narrow down our search window to the region where sigma factors will most likely interact: from 50 bp upstream to 10 bp downstream of the TSS. Indeed, as can be seen in Figure S3, there are many local variations in the bendability and base stacking within this region for the different sigma factors. To confirm if these differences are due to actual variation between sigma factors or simply the result of the large variation of the promoter structural profiles, we performed a two-sided Kolmogorov-Smirnov (KS) test for each sigma factor profile at each position. Based on this analysis, only one of these variations was found to be significant according to a KS-test, namely the stable region in the base stacking profile of sigma 28 at position −14 with a p-value of 1.69 · 10^−5^.

**Figure 4:**
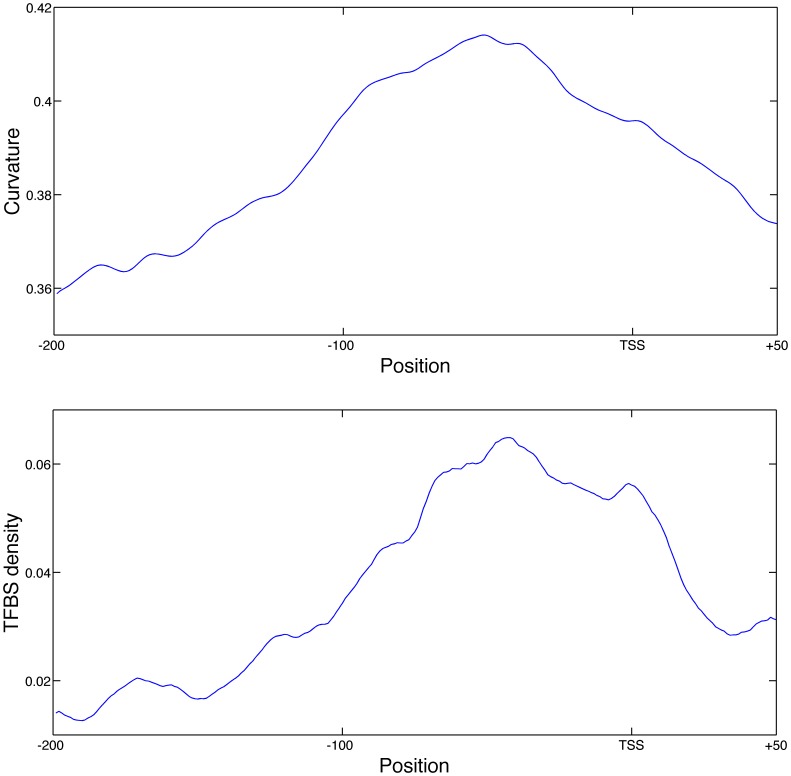
Average *E. coli* promoter curvature and TFBS density profiles. Average structural profiles for *E. coli* promoters from −200 to +50 from the TSS. Top: Average curvature profile of the promoter regions in degrees, where higher values correspond to larger curvature. Bottom: Average transcription factor binding site density profile for the promoter regions, where the values correspond to the frequency of promoters that have a binding site at a given position.

The curvature profiles of the promoters assigned to different sigma factors showed similar results. Again we found differences between the profiles of the various sigma factors but they were not significant. However, as can be seen in Figure S3, the resolution of the curvature profiles is much lower and does not allow a clear evaluation within the scope of functional loci at 60 bp as done for the bendability and base stacking profiles. Figure 4 shows the curvature across the entire promoter region and reveals that the major region of high curvature is not located at the TSS but further upstream. This suggests that there might be limited direct interaction between the functional elements of the core promoter and the DNA curvature. The region starting from about 50 bp upstream from the TSS to about 250 bp upstream, sometimes termed as the proximal promoter, seems to feature more DNA curvature. This is interesting as this region also features many of the binding sites (BS) of transcription factors (TFs). Indeed, comparison of the average curvature profile and the TFBS density in figure 4 reveals highly similar patterns. Thus TFBS seem to occur more in the curved regions of the promoter. When comparing the curvature values of the promoter regions annotated with TFBSs against the promoter regions that have no TFBS annotated, we observed that on average the TFBS have a tendency to occur in regions with a higher curvature (KS-test p-value: 1.87⋅10^−164^). The curvature of the DNA molecule thus co-occurs with the presence of TF binding sites, implying that DNA curvature may play a role in TFBS function. Given this relationship and the diversity in TFs, we evaluated whether there might be a certain subset of TFs that are functionally related to the curvature. If we compare the curvature at the position of all the binding sites of a TF to those of the other TFs, we do indeed identify a set of TF binding sites that occur at significantly high or low curvature, as shown in table 2. We found four TFs whose binding sites have a tendency to occur in curved regions significantly more than other TFs, namely CytR, GlpR, NanR and NhaR, and one which has a lower tendency to occur in curved regions, namely GntR. See the discussion below.

**Table 2:** Transcription factor binding sites with significant average curvature.

Transcription factor	Average curvature	KS-test p-value
CytR	0.70147	2.08E-06
GlpR	0.81358	1.15E-05
NanR	0.64664	0.000485
NhaR	1.0812	0.000162
GntR	0.19353	3.29E-05

Reported are the transcription factors whose binding sites deviated significantly from the promoter mean, the average curvature value of their binding sites and the KS-test p-value.

### The Expression Behavior of Genes can be Linked to Structural Characteristics in Their Promoter Regions

Given the role that the promoter plays in transcription regulation, there might be a direct or indirect link between the groupings of promoters based on their structural profiles and the expression behavior of their downstream genes. To investigate if such a relationship exists, the promoter categories can be compared against the gene expression domains, *i.e.* the conditions under which a gene is up- or downregulated. In a recent study we described three large expression classes in the *Escherichia coli* transcriptome, where each class is a set of genes that share global expression behavior across a large amount of experimental conditions [28]. Detailed analysis of these expression classes showed that each had a clear functional association (see Data S1). One large class (the ‘growth’ class) contained most of the genes that can be associated with housekeeping, essential cellular functions and cell division. A second class of genes (the ‘stress’ class), which displays anti-correlated expression to the growth class, features several genes involved in periods of specific stress or associated alternative metabolism. The last class (the ‘general’ class) displays overlapping expression domains with both the growth and stress classes and includes many of the genes involved in general metabolism and in the mobility of the organism.

**Figure 5:**
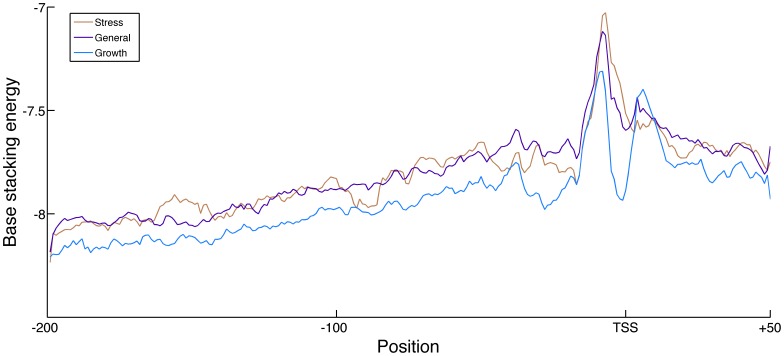
Average promoter stability profiles grouped by expression class. Average base stacking energy structural profiles in kcal/mol of the promoter regions associated with one of the three *E. coli* expression classes, namely the stress class (orange), the general metabolism class (purple) and the growth class (cyan). Higher base stacking energies correspond to lower DNA stability. The structural profiles span 200 nt upstream to 50 nt downstream from the TSS.

Each promoter in our data set can be annotated as being part of one of the three classes based on the functional expression class of the first downstream gene. In this analysis, the complete TSS data set was again analyzed across the larger span of 200 nt downstream to 50 nt upstream. These average profiles are largely similar over the three expression classes with most differences falling well within the expected variation resulting from the original noisy promoter profiles. One exception is a sharp difference between the growth class and the other two classes within the base stacking energy profiles at the region around the TSS, as can be seen in figure 5. The growth class promoters are on average more stable at this position than those from the other classes (KS-test p-value: 6.4⋅10^−11^). Given this large difference in stability and the known correlation between stability and GC content, it is not surprising that the G/C frequency of the promoters of genes in the growth class is higher than that of either the stress or general expression class. Detailed analysis of the base sequence shows that the stability difference at the TSS is caused by a higher preference for guanine and cytosine from minus 5 bp to minus 1 bp relative to the TSS, without any clear pattern for a specific sequence in the growth class promoters (see Figure S4). The GC content or the growth class promoters at position −5 to −1 is 51.2% while that of the general metabolism class and stress class is 41.4% and 36.2%, respectively. This region of higher stability in the growth class promoters is heavily contrasted by the low stability and low GC content at the −10 position and the region downstream from the TSS.

### The Structural Profiles of Proteobacteria are Mostly Dependent on GC Content

**Figure 6:**
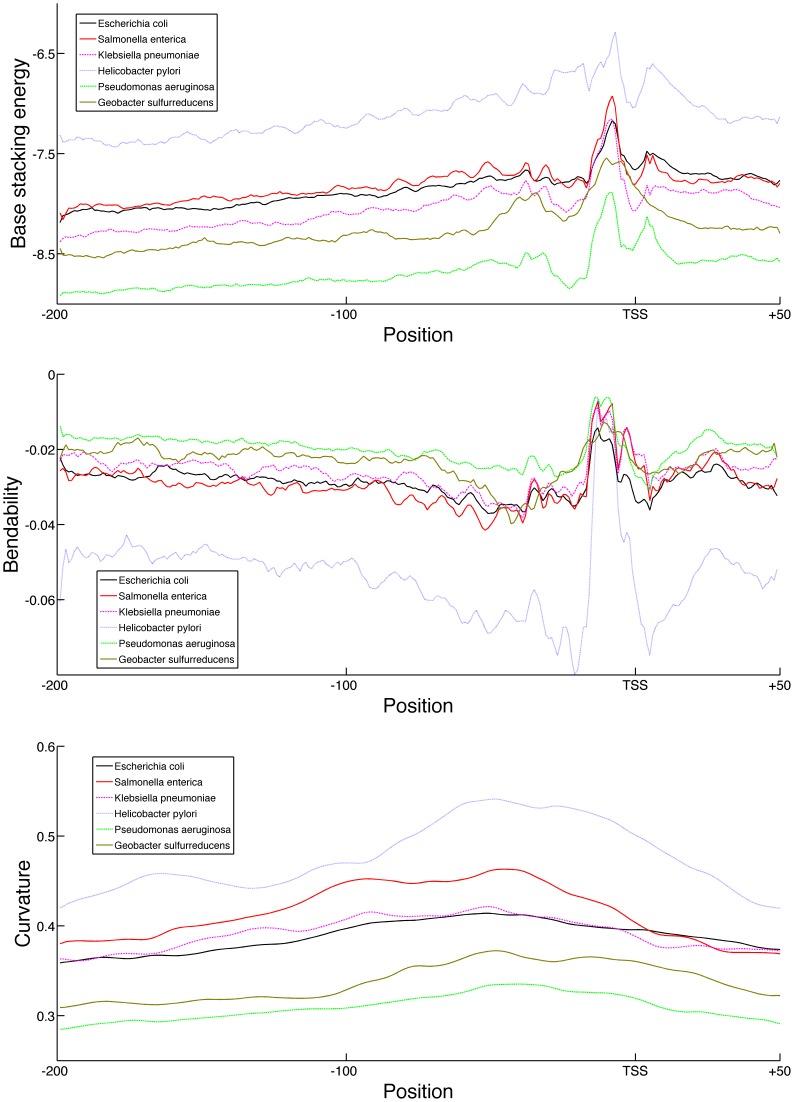
Average structural profiles for six Proteobacteria. Average structural profiles of the promoter regions from *G. sulfurreducens* (solid brown line), *P. aeruginosa* (green dotted line), *S. enterica* (solid red line), *K. pneumoniae* (pink dashed line), *H. pylori* (cyan solid line) and *E. coli* (solid black line). Reported profiles span 200 nt upstream to 50 nt downstream from the TSS. Top: bendability profile as log-frequency where higher values signify more flexible DNA. Middle: Base stacking energy profile in kcal/mol where higher values correspond to less stable DNA. Bottom: Curvature profile in angle degrees where higher values correspond to more curved DNA.

The conservation of the promoter structural profile among related species can be studied by expanding the analysis to other prokaryotes. The Proteobacteria clade, of which *E. coli* is a member, has several species for which large TSS data sets are available: *Klebsiella pneumoniae, Helicobacter pylori*, *Salmonella enterica* serovar Typhimurium, *Pseudomonas aeruginosa* and *Geobacter sulfurreducens*. The Proteobacteria are the largest and most diverse group of gram-negative bacteria. Given the size and number of data sets available for this clade, we will first compare the structural profiles of the Proteobacteria species. Within this phylum, *E. coli*, *S. enterica* and *K. pneumoniae* are Enterobacteria of the Gammaproteobacteria class, while *P. aeruginosa* is a Pseudomonadales of the same class. *H. pylori* belongs to the Epsilonproteobacteria class and *G. sulfurreducens* to the Deltaproteobacteria. As in *E. coli*, the promoters regions were found to be less stable, more rigid and more curved than the genomic background in all species, with the exception of curvature in *H. pylori*. The absolute values of the base stacking energy profiles of the promoter regions, as shown in figure 6, show great variation between the six Proteobacteria. These profiles suggest that the *H. pylori* promoter regions are the least stable, while those of *P. aeruginosa* have the highest stability. This finding coupled with our earlier observation on the correlation between GC-content and base stacking energy suggests that this difference might be caused by the difference in genomic GC-content between this species. Indeed the genomic GC-content of *H. pylori* is the lowest at 39%, while that of *P. aeruginosa* is the highest with 66%. The GC content of the other species is between 50% and 61%. Despite the difference in overall stability, it is interesting to note that all of the Proteobacteria show similar patterns in their base stacking profiles: a very clearly defined unstable −10 region followed by a more stable region around the TSS. The bendability profile showed similar results with the most species following almost the same profile, the only exception being *H. pylori* which features high rigidity. It is however clear from the bendability and stability profiles that the DNA structure surrounding the −10 position has remained mostly conserved in the promoter nucleotide sequence across all the studied species. This conservation is less clear in the promoter consensus sequences (see Figure S7). While the three Enterobacteria and *H. pylori* all share a conserved TA(T/A)AAT consensus sequence at the −10 position, it seems mostly absent in *P. aeruginosa* and *G. sulfurreducens*. Finally, most species also displayed significantly higher curvature values in their promoter regions than in the remainder of their genome. The only exception in this regard is *H. pylori*, which also features extreme curvature regions in the remainder of its genome and thus the high curvature of the promoter region was not found to be significant.

The lack of large expression compendia or sigma factor annotation for these species prevents us from performing equivalent analyses as those performed on *E. coli.* The clear relation that was found between the stability of the TSS region and the functional class of the downstream gene in *E. coli* suggests that gene ontology information could be used to test if such a functional relation also exists in these species. For this test, the genes were extracted with a low base stacking energy value in the base stacking profile at the −4 position of their promoter (the site of maximum difference between the classes in *E. coli*) and their gene ontology enrichment was calculated. This analysis revealed that the promoters of the two Enterobacteria that featured high stability at this position could be linked to several ontology terms. The *K. pneumoniae* genes with a stable region upstream from the TSS were found to be enriched only in processes that were also enriched in the *E. coli* growth functional expression class (as can be found in Data S1), namely carbohydrate derivative metabolic process (p-value of 6.58⋅10^−5^), tRNA processing (p-value of 5.72⋅10^−4^) and ncRNA processing (p-value of 5.72⋅10^−4^). Similar results were found for *S. enterica*, where genes with the stable region were enriched for nucleoside metabolic process (p-value of 4.73⋅10^−4^), organophosphate metabolic process (p-value of 1.76⋅10^−4^), glycosyl compound metabolic process (p-value of 4.73⋅10^−4^) and tRNA modification (p-value of 4.10⋅10^−4^). Again each of these four terms was also enriched in the *E. coli* growth functional expression class, as shown above.

### The Structural Profiles of E. coli are not Representative for all Prokaryotes

The previous analyses were expanded to other non-Protebacteria prokaryotic species for which large TSS data sets are available: *Bacillus anthracis* of the Firmicutes, *Chlamydophila pneumoniae* of the Chlamydiae (also referred to as *Chlamydia pneumoniae*), the cyanobacteria *Synechocystis sp.* and *Synechococcus elongates*, and the archeae *Sulfolobus solfataricus*. All except *S. solfataricus* are bacteria and of these bacteria all except *B. anthracis* are Gram-negative.

**Figure 7:**
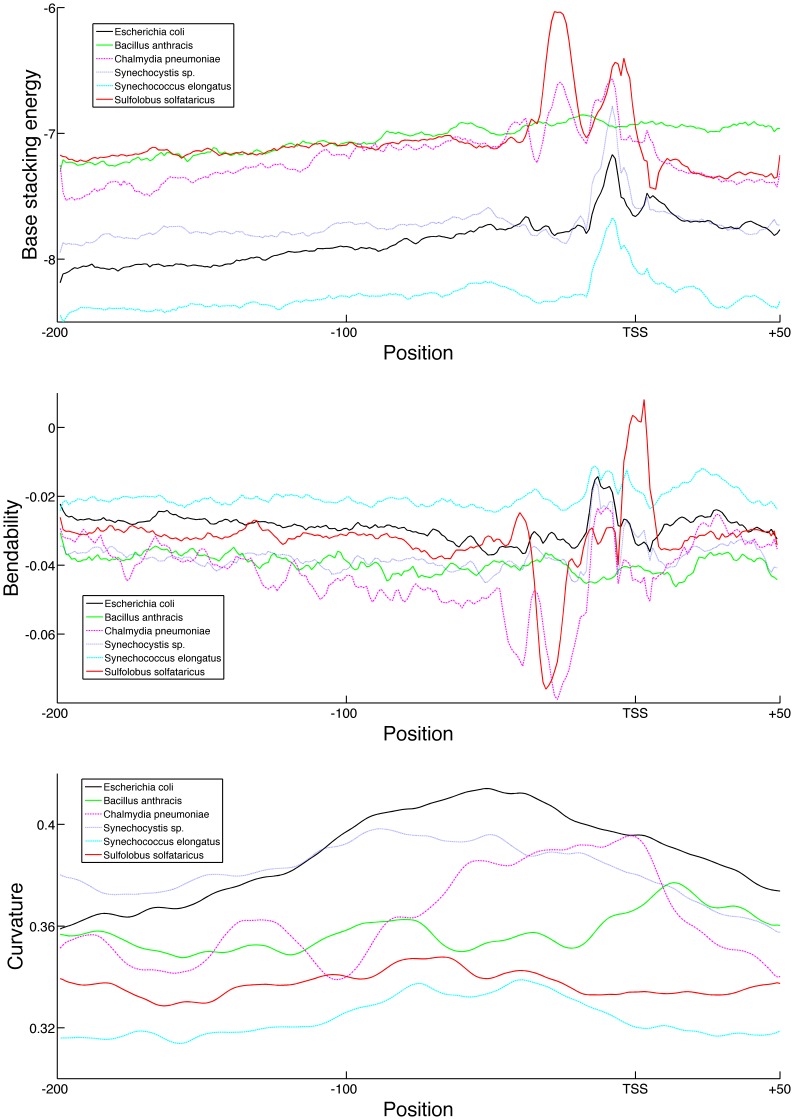
Average structural profiles for six distant prokaryotic species. Average structural profiles of the promoter regions from *B. anthracis* (solid green line), *C. pneumoniae* (pink dotted line), *Synechocystis sp.* (dark blue dotted line), *S. elongatus* (light blue dotted line), *S. solfataricus* (red solid line) and E. coli (solid black line). Reported profiles span 200 nt upstream to 50 nt downstream from the TSS. Top: bendability profile as log-frequency where higher values signify more flexible DNA. Middle: Base stacking energy profile in kcal/mol where higher values correspond to less stable DNA. Bottom: Curvature profile in angle degrees where higher values correspond to more curved DNA.

Again in all cases the promoter regions were found to be significantly less stable and more rigid than the genomic mean. However the promoter regions of the non-Proteobacteria were not found to display higher curvature, with the exception of *C. pneumoniae*. The absolute base stacking values are again correlated to the genomic GC-content of the species, with the highest being 55% for *S. elongates* and the lowest 35% for *B. anthracis*. The base stacking profiles all show extreme values at the −10 position, with the exception of *B. anthracis*, as shown in figure 7. The remainder of the profile for the cyanobacteria is very similar to that previously described for *E. coli*, differing only due to the difference in GC-content. However this conservation around the −10 position is much less clear in the sequence (see Figure S8). The base stacking profile of *C. pneumoniae* and *S. solfataricus* deviate more and feature several additional unstable regions, such as around the −30 position, that were not present in the Proteobacteria. The difference is greater in the bendability profiles, where *S. solfataricus* features a very flexible TSS and a very rigid −30 position. In addition, the bendability profile of *C. pneumoniae* contains a region of high rigidity from −50 to −15 from the TSS.

## Discussion

The structural profiles of promoter regions of six prokaryotic species were studied within the context of their diversity and their function. The main anchor point for these promoter regions were TSS identified through high-throughput experiments or large curated databases. Across all the studied species, the promoter region was found to be less stable and more rigid than the remainder of the genome. The actual structural profiles of the promoter regions were found to differ greatly across the different species, especially at large evolutionary distances. The most consistent pattern was found to be the decreased DNA stability centered around the −10 position across all the Gram-negative bacteria, which likely underlies the success of methods using this structural property for the classification of promoter regions [36]. However the sequence at this position showed more variation in more distant species despite similarities in the structure, suggesting that the some of these structural properties must remain conserved for the function of the promoter even if the sequence is not. This low stability has been suggested to facilitate helix denaturation prior to the transcription event [24]. This pattern was however less clear in the only Gram-positive bacteria tested, namely *B. anthracis*. While this could signify the absence of this pattern in this class of bacteria, it must be noted that this single data set might not be representative for all Gram-positive promoter regions as a whole and/or that the accuracy of TSS determination may not be very high given the absence of regulatory patterns in both the sequence of the structural profiles.

It is interesting to postulate that each sigma factor, which binds near the −10 position and drives the recognition of the promoter region by the RNA polymerase, might have a unique recognition pattern that is present both in the stability or bendability profiles. While some patterns could be found in these structural profiles that are specific to several sigma factors, no clear picture emerged. The correct characterization of these recognition patterns and their statistical significance is likely hampered by interference of several sigma factors binding to the same promoter and limited high-quality data on sigma factor binding. The described patterns also relate to the average structural profiles where the individual promoter profiles display large diversity. Thus any pattern had to be interpreted within large amounts of background noise. Other patterns in the structural profiles extended well beyond the reach of sigma factors, indicating that these might also result from other functional regulatory elements present in the promoter region.

One recurring pattern in the structural profile of promoter region, namely a stable or unstable region at the TSS, could not be linked to any specific sigma factor. This type of pattern could however be associated with either the expression class of the downstream gene or the experimental method used to determine the TSS. Correcting for the patterns distinct for either the expression class or experimental method by plotting the average profiles for the different experimental methods within a single expression class or vice versa, as can be found in Figure S5 and S6, reveals that these are two separate structural patterns. Indeed, as mentioned before the low-high stability region related to the function expression classes is located just ahead of the TSS (from about −5 to −1 upstream), while that from the experimental bias is more concentrated downstream from the TSS (from the TSS itself to about +5 downstream). The explanation behind the presence of these stability regions in either case is not immediately clear from the current study. It should be of note that the pattern described for the growth expression class of a high stability region followed by a sudden dip in stability at the TSS is very similar to the average stability pattern present at the TSS of eukaryotic species, where this stability difference is postulated to aid in correct positioning of the RNA polymerase at the transcription start site [19]. Additionally, the fact that we also found this low and high stability pattern just upstream from the TSS in *K. pneumonia* and *S. enterica* with similar functional annotation enrichment to that found in *E. coli*, not only support the hypothesis that this is a functional element but indicates that it might be conserved throughout the Enterobacteriaceae. However, the lack of homogenized expression data and extensive annotation for these specific species currently limits this analysis. Experiments modifying the stability of the TSS to characterize resulting changes in expression level could provide further insight. Dedicated experiments are indeed the only way to prove that the described structural patterns are in fact functional and not an indirect result from other functional elements, such as those potentially contained in the DNA sequence itself.

Patterns in the DNA curvature profiles diverged strongly from the other structural scales as it is influenced by long range interactions. The findings for the curvature property were not consistent across the studied species; only the promoter regions of some of the Proteobacteria and *C. pneumoniae* were found to have significantly higher curvature values than the remainder of the genome. Additionally, the species that have a higher tendency towards curved promoter regions, only featured high curvature values in typically less than half of their promoters, as was shown for *E. coli*. This implies that high curvature values are not a general characteristic of prokaryotic promoter regions. These regions of high curvature may thus potentially support other types of genomic elements that simply occur in the promoter regions of some species. In the past, the function of the DNA curvature has been postulated to act as a thermosensor or as a functional support for TFBS [39]–[41]. Indeed, we found that regulatory binding sites in *E. coli* were on average more likely to occur in curved regions upstream of the promoter. However detailed analysis showed that while several transcription factor binding sites had a significant tendency to occur in highly curved regions of the promoter, others displayed no preference or seemed even to avoid curved regions. Thus even in species with significantly curved promoters, such as *E. coli*, curved DNA regions are not an absolute requirement for TFBS but likely only specific to a subset of the TFs. Interestingly, the most significant of the high curvature TFs (CytR) and the low curvature TF (GntR) both belong to the LacI-GalR protein family where induction of DNA curvature is known to play a critical role in their regulatory mechanism [42], [43]. CytR is an exception of the LacI-GalR family in this regard, as it cannot induce the required curvature in its target sites and its regulatory effects are dependent on curvature induced by other DNA-binding proteins [44], [45]. The tendency to bind at target sites with high curvature may suggest that CytR could also make use of the intrinsic curvature present in the DNA molecule.

## Conclusions

The structural patterns in prokaryotic promoter regions were re-examined in this study. Despite a lack of data in the past, the estimated structural patterns present at prokaryotic promoter regions obtained by TSS determination seems to have been mostly correct for all tested Gram-negative bacteria; promoter regions are less stable, more rigid and often more curved than genomic DNA. However, large interspecies differences in the structural profiles themselves could be observed. Additionally specific patterns found within the structural profiles of promoters could be linked to the expression behavior of the downstream genes and regulation by sigma factors. The most significant of these findings was a stable/unstable region downstream from the TSS that was associated to the expression class of the downstream gene. These findings have possible implications on promoter prediction tools that use structural properties, as they may be biased towards a certain type of promoter based on sigma factor recognition or a single gene expression class. Finally this work offers new prospects for future research into yet uncharacterized functional elements that are defined by DNA structural properties.

## Materials and Methods

### Data Sets

An overview of the TSS data utilized in this study is given in table 3. The TSS determined for *E. coli* were grouped together into a single data set. To compile a complete set of all TSS from these different data sources without biases by including the same region more than once, we choose to limit our analysis to one TSS per gene. The TSS chosen was always the furthest upstream TSS that had been found from the gene, the rationale being that these TSSs are the least likely to be a false positive, e.g. 5′ end of a processed transcript.

**Table 3:** Transcription start site data used in this study.

Data	Organism	Total TSS	High throughput	5′-end enrichment
PromEC curation [51]	*E. coli*	373		
RegulonDB curation [47]	*E. coli*	1372		
Cho *et al.* [52]	*E. coli*	3475	X	
Kim *et al.* [53]	*E. coli*	2773	X	X
Mendoza-Vargas *et al.* [54]	*E. coli*	208	X	
Mendoza-Vargas *et al.* [54]	*E. coli*	902	X	
Gama-Castro *et al.* [55]	*E. coli*	1309	X	X
Salgado *et al.* [47]	*E. coli*	2731	X	/
Kim *et al.* [53]	*K. pneumoniae*	1597	X	X
Sharma *et al.* [25]	*H. plylori*	636	X	X
Wurtzel *et al.* [56]	*P. aeruginosa*	2117	X	X
Kroger *et al.* [57]	*S. enterica*	1192	X	X
Qiu *et al.* [58]	*G. sulfurreducens*	1063	X	X
Albrecht *et al.* [59]	*C. pneumoniae*	376	X	X
Passalacqua *et al.* [60]	*B. antracis*	807	X	
Mitschke *et al.* [61]	*Synechocystis sp.*	1098	X	X
Vijayan *et al.* [62]	*S. elongates*	1473	X	
Wurtzel *et al.* [63]	*S. solfataricus*	1052	X	X

The DNA sequences were derived from the genome information available at NCBI for the specific strain for which the TSS was determined, namely E. coli K12 (NC_000913), K. pneumoniae MGH 78578 (NC_009648), H. pylori 26695 (NC_000915), G. sulfurreducens (NC_002939.5), S. enterica SL1344 (NC_016810), P. aeruginosa PA14 (NC_008463.1), B. anthracis str. Sterne (NC_005945), Synechocystis sp. PCC 6803 (NC_000911.1), S. elongatus PCC 7942 (NC_007604.1), S. solfataricus P2 (NC_002754.1) and C. pneumoniae CWL029 (NC_000922) [46]. The sequence region used for the structural profile analysis was then either 200 nt upstream to 50 nt downstream from the TSS, or 50 nt upstream to 10 nt downstream from the reported TSS (see below).

For the comparison of the data sources, we compiled a set of those found by low-throughput methods (RegulonDB and PromEC curated lists), HT methods without preprocessing steps (RACE by Cho et al., two experiments by Mendoza-Vargas et al.) and HT experiments with enrichment for tri-phosphate-capped RNA (RACE by Kim at al., RNAseq by Gama-Castro et al.). The RNAseq results detailed in Salgado *et al.* (2013) was not included in the experimental compilation as it represents a mixture between a high-throughput method with and without preprocessing [47], however it is included in the complete *E. coli* TSS data set.

Sigma factor and transcription factor binding sites were obtained from the annotations in RegulonDB (version 7.5) supported by strong evidence. Enrichment calculations were performed based on a hypergeometric distribution. The likelihood that two samples are derived from the same distribution was evaluated based on a two-sided Kolmogorov-Smirnov (KS) test. Note we apply the KS-test for two types of analyses in this study. The first is a statistical comparison between distributions of mean values for entire sequences, e.g. in the comparative analysis of average structural values between promoter and non-promoter sequences. The second is a screening of an entire profile to identify positions where the profile differs significantly from a background. In this case the two distributions that are compared are that of the structural values at a certain position within a specific subset of promoter regions, e.g. those associated with a sigma factor or a gene expression class, and the structural values at the same position in all remaining promoter regions of the same species. Both the hypergeometric and KS testing were performed using the tools available within Matlab 2013a. The resulting p-values were evaluated for significance at a threshold of 0.05 divided by the number of tests performed within the experiment as per the Bonferroni correction.

### Structural Profiles

The structural properties used in this manuscript were derived from experiments or theoretical approaches and stored in structural scales. The structural scale model is based on the neighbor model of DNA structure, which postulates that the primary structural characteristics of the DNA molecule are the result of neighboring base interactions. As such the structure of a DNA molecule can be derived with reasonable accuracy from the sequence if the contributions of each di- or trinucleotide to the overall structure are known. The structural scales detail this contribution for each di−/trinucleotide. The structural scales can be applied to any DNA sequence to calculate the structural profile, a vector listing the contribution of each di−/tri-nucleotide in the sequence at the respective position. This profile can readily be used for study of the DNA structure, except for the curvature calculation requiring additional steps (see below). In all cases discussed here however, a loess-smoothing with range 10 nt was applied. When dealing with average profiles, the smoothing was done prior to the calculation of the average.

Base stacking energy is a dinucleotide scale that was derived from the theoretical calculations of Ornstein *et al.* [30]. This scale contains the minimal free energy, as expressed by kilocalories/mol, for each dinucleotide. The more negative this energy is, the more stable the base stacking and thus the DNA helix is expected to be.

Denaturation temperature is a dinucleotide scale derived from the experimental observations of Delcourt and Blake [48]. This scale gives for each dinucleotide the average melting temperature in °C. Thus the higher this temperature is, the more stable the DNA molecule.

B-DNA twist is derived from the observations made by Olson *et al.* [1] and provides the average angle found between successive base pairs for each dinucleotide. The reported values are the twist angle in degrees between successive base pairs. It has been observed that this scale is a good measure for the rigidity of the DNA molecule, as more rigid DNA molecules tend to have a higher twist [33].

Z-DNA-philicity is a dinucleotide scale derived from the calculations by Ho *et al.* [34], who determined the free energy of the dinucleotide if present in the Z-DNA conformation expressed in kilocalories/mol. Here sequences with lower values will be more inclined to be in the Z-DNA conformation.

Major groove bendability is a trinucleotide scale derived from the DNase-I cutting frequencies as defined by Brukner *et al.* [35]. The reported values are the log of these cutting frequencies, i.e. the less a sequence is cut by DNAase-I, the more negative the assigned value will be. As DNase-I will cut a DNA molecule when it is bent towards the major groove, sequences with high scores in this scale will be more likely to have an intrinsic bend towards the major groove or to be flexible in this direction.

Curvature is calculated in the manner of the BEND algorithm as detailed by Goodsell and Dickerson [32]. Here curvature is defined as the sum of the local additive intrinsic deformation of the different base pairs along the helical axis. Its calculation is based on three dinucleotide scales, derived from the original BEND algorithm, for the twist, tilt and roll of the DNA molecule. This information is used to calculate the average trajectory of the DNA axis over 10 base pairs, which is then used to derive the average curvature of each position based on the trajectory 50 base pairs up- and downstream. The reported values are thus the relative angle of the DNA curvature in degrees across these 100 bp. Due to the long range nature of curvature defined in this manner, the curvature was first calculated for the entire genome and then assigned to each separate sequence based on its location.

Smoothing of the structural properties is performed by applying a loess regression to the numerical vector derived from the structural scales after application on the DNA sequence. Unless indicated otherwise, all structural profiles were smoothed with a span of 10 nt.

### Non-structural Profiles

The GC content profiles are derived from a scale where each dinucleotide is assigned a value identical to the number of strong bases it contains (e.g. AT is assigned a 0, AG is assigned a 1, GC is assigned a 2, etc.). While it is not a true structural scale, it is well known that the GC content influences many aspects of the DNA molecular structure. However the reported GC content frequencies, e.g. in the cross-species and functional gene expression class analyses, are not derived directly from this scale but are simply the percentage of either G or C in the corresponding genomic sequences.

The transcription factor binding sites density was calculated by assigning a value of ‘one’ to each genomic position where a transcription factor is reported to bind according to the data available in RegulonDB and ‘zero’ if not [47]. The average profile over several sequences can therefore be interpreted as the frequency of binding sites across all sequences at a specific position.

### Functional Gene Expression Classes

The expression classes are derived from the large *E. coli* expression compendia found in COLOMBOS (release 2, June 2012) [27], [49]. Correlation matrices were calculated for all genes in the compendia, where each element_ij_ is the correlation of the gene_i_ versus gene_j_ over all conditions. A hierarchical clustering method was then applied which identified three large categories of genes present in the genome. These three expression classes could then be related to three general biological states: survival in stressful conditions, growth and general metabolism. More information about the nature and construction of these clusters can be found in Meysman et al. [28]. Gene ontology enrichments of these three classes can be found in Data S1.

### Gene Ontology Enrichment of the Stability Profiles

To analyze the functional relationship between the stability of the TSS region and the functional annotation of the downstream genes, the TSSs with low stability at the −4 position were extracted from the collection of structural profiles for each species. The cut-off for ‘low stability’ was determined based on the distribution of the base stacking values at this position and differed based on the GC content. The cut-off for the Enterobacteriaceae was set at −8.5, while those of the other more AT-rich species was set at −7.5. The extracted TSSs were then mapped unto the first gene in their associated operon. The functional annotation of the resulting gene list was then evaluated based on the gene ontology annotation available in UniProtKB-GOA (accessed on February 15, 2013). Enrichment for all gene ontology terms mapped to these genes and all of the ancestor ontology terms were estimated using a hypergeometric distribution. The background set for comparison consisted of all the genes downstream from a described TSS with gene ontology annotation. A p-value cut-off of 0.1, corrected for multiple testing with a Bonferonni approach by dividing by the number of tested ontology terms, was used for significance.

## Supporting Information

Figure S1 **Motif logos generated with WebLogo** [**50**] **of the promoter sequences from 50 bp upstream to 10 bp downstream from the TSS.** Each set is grouped by the experimental method used to determine the TSS, namely curated low-throughput methods, high-throughput methods without enrichment procedures, and high-throughput methods preceded by an enrichment step for primary transcripts.
(PDF)

Figure S2 **Average curvature profiles of four groups of clustered** ***E. coli*** **promoters.** The clustering was achieved by using the k-means algorithm as available in Matlab 2013a on the curvature profiles of all *E. coli* promoters in our data set. The number of clusters was fixed at four and thus the promoters were grouped together in four groups based on similarities in their curvature profiles, termed Cluster 1 (387 promoters), Cluster 2 (1174 promoters), Cluster 3 (327 promoters) and Cluster 4 (318 promoters).
(PDF)

Figure S3 **Average structural profiles for promoters annotated to be regulated by a given sigma factor smoothed with a span of 5.** Top: Average bendability profiles for promoters annotated to be regulated by the given sigma factor with high values corresponding to higher flexibility. Middle: Average base stacking profiles for promoters annotated to be regulated by the given sigma factor with higher values corresponding to lower stability. Bottom: Average curvature profiles for promoters annotated by the given sigma factor where higher values correspond to more curvature.
(PDF)

Figure S4 **Motif logos generated with WebLogo** [**50**] **of the promoter sequences from 50 bp upstream to 10 bp downstream from the TSS.** Each set is grouped by the expression class of the first downstream gene, namely the stress expression class, general metabolism expression class, and growth expression class.
(PDF)

Figure S5 **Average base stacking profiles of the three expression classes grouped by experimental method used to determine the TSS.**
(PDF)

Figure S6 **Average base stacking energy profiles of the three experimental categories used to determine the TSS location grouped by functional expression class.**
(PDF)

Figure S7 **Motif logos generated with WebLogo** [**50**] **of the promoter regions (50 bp upstream to 10 bp downstream the TSS) from the six studied Proteobacteria.**
(PDF)

Figure S8 **Motif logos generated with WebLogo** [**50**] **of the promoter regions (50 bp upstream to 10 bp downstream the TSS) from the six studied species from different phyla.**
(PDF)

Data S1 **Gene ontology enrichment results for each of the three expression classes compared against the set of all genes for which expression data is available.**
(PDF)

## References

[1] Olson WK, Gorin AA, Lu XJ, Hock LM, Zhurkin VB (1998) DNA sequence-dependent deformability deduced from protein-DNA crystal complexes. Proc Natl Acad Sci U S A 95: 11163–11168. 9736707 10.1073/pnas.95.19.11163 PMC21613

[2] Baisnée PF, Baldi P, Brunak S, Pedersen AG (2001) Flexibility of the genetic code with respect to DNA structure. Bioinformatics 17: 237–248. 11294789 10.1093/bioinformatics/17.3.237

[3] Meysman P, Marchal K, Engelen K (2012) DNA structural properties in the classification of genomic transcription regulation elements. Bioinform Biol Insights 6: 155–168. 22837642 10.4137/BBI.S9426 PMC3399529

[4] Baldi P, Chauvin Y, Brunak S, Gorodkin J, Pedersen a G (1998) Computational applications of DNA structural scales. Proc Int Conf Intell Syst Mol Biol 6: 35–42. 9783207

[5] Friedel M, Nikolajewa S, Sühnel J, Wilhelm T (2009) DiProDB: a database for dinucleotide properties. Nucleic Acids Res 37: D37–40. 18805906 10.1093/nar/gkn597 PMC2686603

[6] Packer MJ, Dauncey MP, Hunter C a (2000) Sequence-dependent DNA structure: dinucleotide conformational maps. J Mol Biol 295: 71–83. 10623509 10.1006/jmbi.1999.3236

[7] Pedersen AG, Jensen LJ, Brunak S, Staerfeldt HH, Ussery DW (2000) A DNA structural atlas for Escherichia coli. J Mol Biol 299: 907–930. 10843847 10.1006/jmbi.2000.3787

[8] Abeel T, Saeys Y, Rouzé P, Van de Peer Y (2008) ProSOM: core promoter prediction based on unsupervised clustering of DNA physical profiles. Bioinformatics 24: i24–31. 18586720 10.1093/bioinformatics/btn172 PMC2718650

[9] Kanhere A, Bansal M (2005) Structural properties of promoters: similarities and differences between prokaryotes and eukaryotes. Nucleic Acids Res 33: 3165–3175. 15939933 10.1093/nar/gki627 PMC1143579

[10] Gabrielian A, Bolshoy A (1999) Sequence complexity and DNA curvature. Comput Chem 23: 263–274. 10404619 10.1016/s0097-8485(99)00007-8

[11] Parbhane RV, Tambe SS, Kulkarni BD (2000) ANN modeling of DNA sequences: new strategies using DNA shape code. Comput Chem 24: 699–711. 10966128 10.1016/s0097-8485(00)00072-3

[12] Conilione P, Wang D (2005) Neural Classification of E.coli Promoters Using Selected DNA profiles. Proceedings of the fourth IEEE International Workshop WSTST ’ 05: 51–60.

[13] Mallios RR, Ojcius DM, Ardell DH (2009) An iterative strategy combining biophysical criteria and duration hidden Markov models for structural predictions of Chlamydia trachomatis sigma66 promoters. BMC Bioinformatics 10: 271. 19715597 10.1186/1471-2105-10-271 PMC2743672

[14] Meysman P, Dang TH, Laukens K, De Smet R, Wu Y, et al (2010) Use of structural DNA properties for the prediction of transcription-factor binding sites in Escherichia coli. Nucleic Acids Res 39: 1–11. 21051340 10.1093/nar/gkq1071 PMC3025552

[15] Fernandez M, Fujii S, Kono H, Sarai A (2009) Evaluation of DNA intramolecular interactions for nucleosome positioning in yeast. Genome Inform 23: 13–20. 20180258

[16] Geurts AM, Hackett CS, Bell JB, Bergemann TL, Collier LS, et al (2006) Structure-based prediction of insertion-site preferences of transposons into chromosomes. Nucleic Acids Res 34: 2803–2811. 16717285 10.1093/nar/gkl301 PMC1464413

[17] Ohler U, Niemann H, Liao Gc, Rubin GM (2001) Joint modeling of DNA sequence and physical properties to improve eukaryotic promoter recognition. Bioinformatics 17 Suppl 1: S199–206. 11473010 10.1093/bioinformatics/17.suppl_1.s199

[18] Goñi JR, Pérez A, Torrents D, Orozco M (2007) Determining promoter location based on DNA structure first-principles calculations. Genome Biol 8: R263. 18072969 10.1186/gb-2007-8-12-r263 PMC2246265

[19] Gan Y, Guan J, Zhou S (2009) A pattern-based nearest neighbor search approach for promoter prediction using DNA structural profiles. Bioinformatics 25: 2006–2012. 19515962 10.1093/bioinformatics/btp359

[20] Morey C, Mookherjee S, Rajasekaran G, Bansal M (2011) DNA free energy-based promoter prediction and comparative analysis of Arabidopsis and rice genomes. Plant Physiol 156: 1300–1315. 21531900 10.1104/pp.110.167809 PMC3135951

[21] Florquin K, Saeys Y, Degroeve S, Rouzé P, Van de Peer Y (2005) Large-scale structural analysis of the core promoter in mammalian and plant genomes. Nucleic Acids Res 33: 4255–4264. 16049029 10.1093/nar/gki737 PMC1181242

[22] Wang H, Benham CJ (2006) Promoter prediction and annotation of microbial genomes based on DNA sequence and structural responses to superhelical stress. BMC Bioinformatics 7: 248. 16677393 10.1186/1471-2105-7-248 PMC1468432

[23] Askary A, Masoudi-Nejad A, Sharafi R, Mizbani A, Parizi SN, et al (2009) N4: a precise and highly sensitive promoter predictor using neural network fed by nearest neighbors. Genes Genet Syst 84: 425–430. 20228580 10.1266/ggs.84.425

[24] Rangannan V, Bansal M (2009) Relative stability of DNA as a generic criterion for promoter prediction: whole genome annotation of microbial genomes with varying nucleotide base composition. Mol Biosyst 5: 1758–1769. 19593472 10.1039/B906535K

[25] Sharma CM, Hoffmann S, Darfeuille F, Reignier J, Findeiss S, et al (2010) The primary transcriptome of the major human pathogen Helicobacter pylori. Nature 464: 250–255. 20164839 10.1038/nature08756

[26] Evguenieva-Hackenberg E, Klug G (2011) New aspects of RNA processing in prokaryotes. Curr Opin Microbiol 14: 587–592. 21945217 10.1016/j.mib.2011.07.025

[27] Meysman P, Sonego P, Bianco L, Fu Q, Ledezma-Tejeida D, et al. (2013) COLOMBOS v2.0: An ever expanding collection of bacterial expression compendia. Nucleic Acids Res: gkt1086. 10.1093/nar/gkt1086 PMC3965013 24214998

[28] Meysman P, Sanchez-Rodríguez A, Fu Q, Marchal K, Engelen K (2013) Expression divergence between Escherichia coli and Salmonella enterica serovar Typhimurium reflects their lifestyles. Mol Biol Evol 30: 1302–1314. 23427276 10.1093/molbev/mst029 PMC3649669

[29] Madan Babu M, Teichmann SA (2003) Functional determinants of transcription factors in Escherichia coli: protein families and binding sites. Trends Genet 19: 75–79. 12547514 10.1016/S0168-9525(02)00039-2

[30] Ornstein RL, Rein R, Breen DL, Macelroy RD (1978) An optimized potential function for the calculation of nucleic acid interaction energies I. Base stacking. Biopolymers 17: 2341–2360. 24624489 10.1002/bip.1978.360171005

[31] Delcourt S, Blake R (1991) Stacking energies in DNA. J Biol Chem 266: 15160–15169. 1869547

[32] Goodsell DS, Dickerson RE (1994) Bending and curvature calculations in B-DNA. Nucleic Acids Res 22: 5497–5503. 7816643 10.1093/nar/22.24.5497 PMC332108

[33] Gorin AA, Zhurkin VB (1995) B-DNA Twisting Correlates with Base-pair Morphology. J Mol Biol 247: 34–48. 7897660 10.1006/jmbi.1994.0120

[34] Ho PS, Ellison MJ, Quigley GJ, Rich A (1986) A computer aided thermodynamic approach for predicting the formation of Z-DNA in naturally occurring sequences. EMBO J 5: 2737–2744. 3780676 10.1002/j.1460-2075.1986.tb04558.x PMC1167176

[35] Brukner I, Sánchez R, Suck D, Pongor S (1995) Sequence-dependent bending propensity of DNA as revealed by DNase I: parameters for trinucleotides. EMBO J 14: 1812–1818. 7737131 10.1002/j.1460-2075.1995.tb07169.x PMC398274

[36] Kanhere A, Bansal M (2005) A novel method for prokaryotic promoter prediction based on DNA stability. BMC Bioinformatics 6: 1. 15631638 10.1186/1471-2105-6-1 PMC545949

[37] Baldi P, Baisnée PF (2000) Sequence analysis by additive scales: DNA structure for sequences and repeats of all lengths. Bioinformatics 16: 865–889. 11120677 10.1093/bioinformatics/16.10.865

[38] Wosten MMSM (1998) Eubacterial sigma-factors. FEMS Microbiol Rev 22: 127–150. 9818380 10.1111/j.1574-6976.1998.tb00364.x

[39] Nov Klaiman T, Hosid S, Bolshoy A (2009) Upstream curved sequences in E. coli are related to the regulation of transcription initiation. Comput Biol Chem 33: 275–282. 19646927 10.1016/j.compbiolchem.2009.06.007

[40] Prosseda G, Falconi M, Giangrossi M, Gualerzi CO, Micheli G, et al (2004) The virF promoter in Shigella: more than just a curved DNA stretch. Mol Microbiol 51: 523–537 doi:–10.1046/j.1365–2958.2003.03848.x 14756791 10.1046/j.1365-2958.2003.03848.x

[41] Olivares-Zavaleta N, Jáuregui R, Merino E (2006) Genome analysis of Escherichia coli promoter sequences evidences that DNA static curvature plays a more important role in gene transcription than has previously been anticipated. Genomics 87: 329–337. 16413165 10.1016/j.ygeno.2005.11.023

[42] Kalodimos CG, Boelens R, Kaptein R (2004) Toward an integrated model of protein-DNA recognition as inferred from NMR studies on the Lac repressor system. Chem Rev 104: 3567–3586. 15303828 10.1021/cr0304065

[43] Spronk CA, Folkers GE, Noordman AM, Wechselberger R, van den Brink N, et al (1999) Hinge-helix formation and DNA bending in various lac repressor-operator complexes. EMBO J 18: 6472–6480. 10562559 10.1093/emboj/18.22.6472 PMC1171710

[44] Kallipolitis BH, Nørregaard-Madsen M, Valentin-Hansen P (1997) Protein-protein communication: structural model of the repression complex formed by CytR and the global regulator CRP. Cell 89: 1101–1109. 9215632 10.1016/s0092-8674(00)80297-4

[45] Meysman P, Marchal K, Engelen K (2013) Identifying common structural DNA properties in transcription factor binding site sets of the LacI-GalR family. Curr Bioinform 8: 483–488.

[46] Pruitt KD, Tatusova T, Brown GR, Maglott DR (2012) NCBI Reference Sequences (RefSeq): current status, new features and genome annotation policy. Nucleic Acids Res 40: D130–5. 22121212 10.1093/nar/gkr1079 PMC3245008

[47] Salgado H, Peralta-Gil M, Gama-Castro S, Santos-Zavaleta A, Muñiz-Rascado L, et al (2013) RegulonDB v8.0: omics data sets, evolutionary conservation, regulatory phrases, cross-validated gold standards and more. Nucleic Acids Res 41: D203–D213. 23203884 10.1093/nar/gks1201 PMC3531196

[48] Breslauer KJ, Frank R, Blocker H, Marky LA (1986) Predicting DNA Duplex Stability from the Base Sequence. Proc Natl Acad Sci 83: 3746–3750. 3459152 10.1073/pnas.83.11.3746 PMC323600

[49] Engelen K, Fu Q, Meysman P, Sánchez-Rodríguez A, De Smet R, et al (2011) COLOMBOS: Access Port for Cross-Platform Bacterial Expression Compendia. PLoS One 6: e20938. 21779320 10.1371/journal.pone.0020938 PMC3136457

[50] Crooks GE, Hon G, Chandonia J-M, Brenner SE (2004) WebLogo: a sequence logo generator. Genome Res 14: 1188–1190. 15173120 10.1101/gr.849004 PMC419797

[51] Hershberg R, Bejerano G, Santos-Zavaleta A, Margalit H (2001) PromEC: An updated database of Escherichia coli mRNA promoters with experimentally identified transcriptional start sites. Nucleic Acids Res 29: 277. 11125111 10.1093/nar/29.1.277 PMC29777

[52] Cho B, Zengler K, Qiu Y, Park YS, Knight EM, et al (2009) The transcription unit architecture of the Escherichia coli genome. Nat Biotechnol 27: 1043–1049. 19881496 10.1038/nbt.1582 PMC3832199

[53] Kim D, Hong JS-J, Qiu Y, Nagarajan H, Seo J-H, et al (2012) Comparative Analysis of Regulatory Elements between Escherichia coli and Klebsiella pneumoniae by Genome-Wide Transcription Start Site Profiling. PLoS Genet 8: e1002867. 22912590 10.1371/journal.pgen.1002867 PMC3415461

[54] Mendoza-Vargas A, Olvera L, Olvera M, Grande R, Vega-Alvarado L, et al (2009) Genome-wide identification of transcription start sites, promoters and transcription factor binding sites in E. coli. PLoS One 4: e7526. 19838305 10.1371/journal.pone.0007526 PMC2760140

[55] Gama-Castro S, Salgado H, Peralta-Gil M, Santos-Zavaleta A, Muñiz-Rascado L, et al (2011) RegulonDB version 7.0: transcriptional regulation of Escherichia coli K-12 integrated within genetic sensory response units (Gensor Units). Nucleic Acids Res 39: D98–105. 21051347 10.1093/nar/gkq1110 PMC3013702

[56] Wurtzel O, Yoder-Himes DR, Han K, Dandekar AA, Edelheit S, et al (2012) The single-nucleotide resolution transcriptome of Pseudomonas aeruginosa grown in body temperature. PLoS Pathog 8: e1002945. 23028334 10.1371/journal.ppat.1002945 PMC3460626

[57] Kröger C, Dillon SC, Cameron ADS, Papenfort K, Sivasankaran SK, et al (2012) The transcriptional landscape and small RNAs of Salmonella enterica serovar Typhimurium. Proc Natl Acad Sci U S A 109: E1277–86. 22538806 10.1073/pnas.1201061109 PMC3356629

[58] Qiu Y, Cho B, Park YS, Lovley D, Palsson BØ, et al (2010) Structural and operational complexity of the Geobacter sulfurreducens genome. Genome Res 20: 1304–1311 doi:10.1101/gr.107540.110 20592237 10.1101/gr.107540.110 PMC2928509

[59] Albrecht M, Sharma CM, Dittrich MT, Müller T, Reinhardt R, et al (2011) The transcriptional landscape of Chlamydia pneumoniae. Genome Biol 12: R98. 21989159 10.1186/gb-2011-12-10-r98 PMC3333780

[60] Passalacqua KD, Varadarajan A, Ondov BD, Okou DT, Zwick ME, et al (2009) Structure and complexity of a bacterial transcriptome. J Bacteriol 191: 3203–3211. 19304856 10.1128/JB.00122-09 PMC2687165

[61] Mitschke J, Georg J, Scholz I, Sharma CM, Dienst D, et al (2011) An experimentally anchored map of transcriptional start sites in the model cyanobacterium Synechocystis sp. PCC6803. Proc Natl Acad Sci U S A 108: 2124–2129. 21245330 10.1073/pnas.1015154108 PMC3033270

[62] Vijayan V, Jain IH, O’Shea EK (2011) A high resolution map of a cyanobacterial transcriptome. Genome Biol 12: R47. 21612627 10.1186/gb-2011-12-5-r47 PMC3219970

[63] Wurtzel O, Sapra R, Chen F, Zhu Y, Simmons BA, et al (2010) A single-base resolution map of an archaeal transcriptome. Genome Res 20: 133–141. 19884261 10.1101/gr.100396.109 PMC2798825

