# Supplementary material for: Structural Properties of Prokaryotic Promoter Regions Correlate with Functional Features
Source: PLoS One. 2014 Feb 7;9(2):e88717. doi: 10.1371/journal.pone.0088717 (PMC3918002; doi:10.1371/journal.pone.0088717)
Supplement: Figure S1 — Motif logos generated with WebLogo [50] of the promoter sequences from 50 bp upstream to 10 bp downstream from the TSS. Each set is grouped by the experimental method used to determine the TSS, namely curated low-throughput methods, high-throughput methods without enrichment procedures, and high-throughput methods preceded by an enrichment step for primary transcripts. (PDF) [file pone.0088717.s001.pdf]

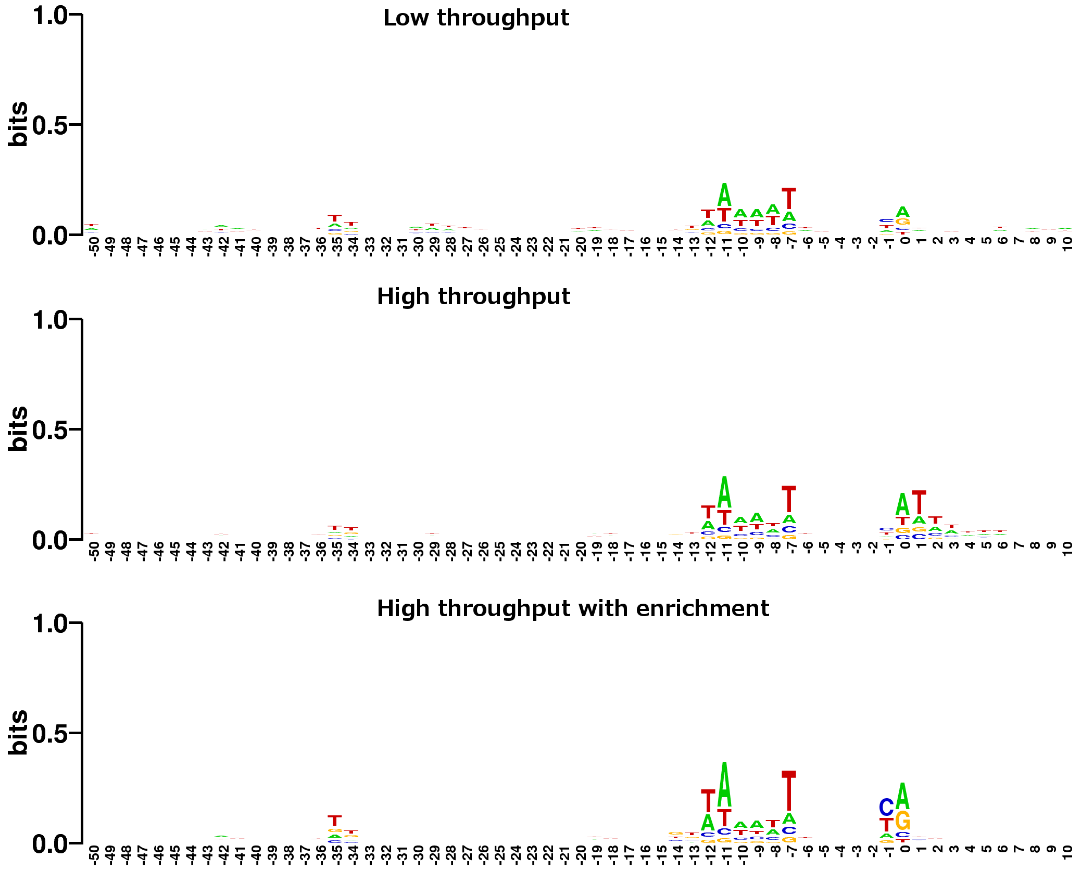

Supplemental figure S1: Motif logos generated with WebLogo of the promoter sequences from 50bp upstream to 10bp downstream from the TSS. Each set is grouped by the experimental method used to determine the TSS, namely curated low-throughput methods (top), high-throughput methods without enrichment procedures (middle) and high-throughput methods preceded by an enrichment step for primary transcripts (bottom). Note the y-axis only covers 1 bit instead of the standard 2 bits for motif logos, so that the very degenerate consensus sequences are still legible.
