# Supplementary material for: Structural Properties of Prokaryotic Promoter Regions Correlate with Functional Features
Source: PLoS One. 2014 Feb 7;9(2):e88717. doi: 10.1371/journal.pone.0088717 (PMC3918002; doi:10.1371/journal.pone.0088717)
Supplement: Figure S2 — Average curvature profiles of four groups of clustered E. coli promoters. The clustering was achieved by using the k-means algorithm as available in Matlab 2013a on the curvature profiles of all E. coli promoters in our data set. The number of clusters was fixed at four and thus the promoters were grouped together in four groups based on similarities in their curvature profiles, termed Cluster 1 (387 promoters), Cluster 2 (1174 promoters), Cluster 3 (327 promoters) and Cluster 4 (318 promoters). (PDF) [file pone.0088717.s002.pdf]

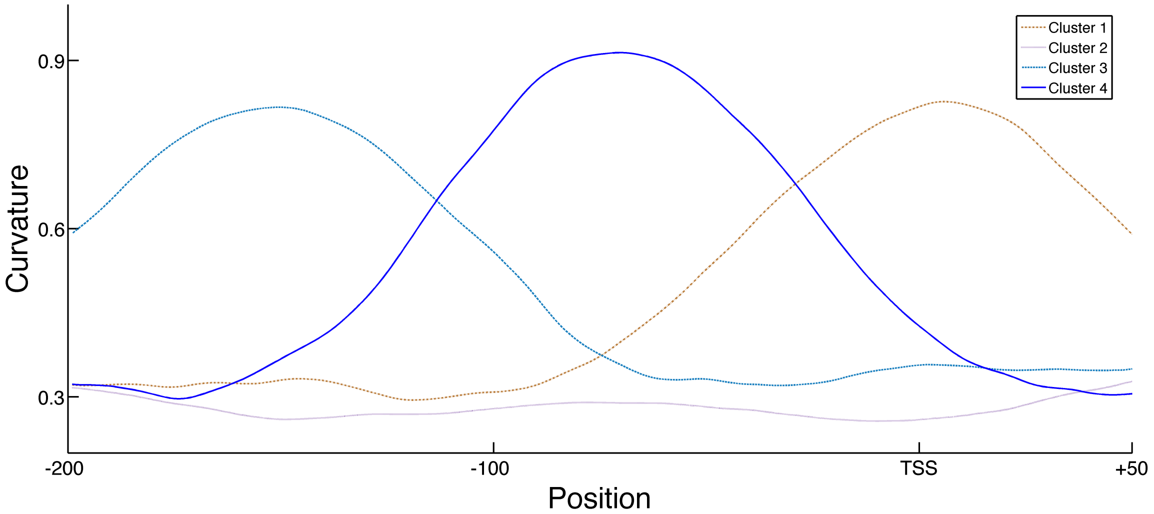

Supplemental figure S2: Average curvature profiles of four groups of clustered *E. coli* promoters. The clustering was achieved by using the k-means algorithm as available in Matlab 2013a on the curvature profiles of all *E. coli* promoters in our data set. The number of clusters was fixed at four and thus the promoters were grouped together in four groups based on similarities in their curvature profiles, termed Cluster 1 (387 promoters), Cluster 2 (1174 promoters), Cluster 3 (327 promoters) and Cluster 4 (318 promoters).
