# Supplementary material for: Structural Properties of Prokaryotic Promoter Regions Correlate with Functional Features
Source: PLoS One. 2014 Feb 7;9(2):e88717. doi: 10.1371/journal.pone.0088717 (PMC3918002; doi:10.1371/journal.pone.0088717)
Supplement: Figure S3 — Average structural profiles for promoters annotated to be regulated by a given sigma factor smoothed with a span of 5. Top: Average bendability profiles for promoters annotated to be regulated by the given sigma factor with high values corresponding to higher flexibility. Middle: Average base stacking profiles for promoters annotated to be regulated by the given sigma factor with higher values corresponding to lower stability. Bottom: Average curvature profiles for promoters annotated by the given sigma factor where higher values correspond to more curvature. (PDF) [file pone.0088717.s003.pdf]

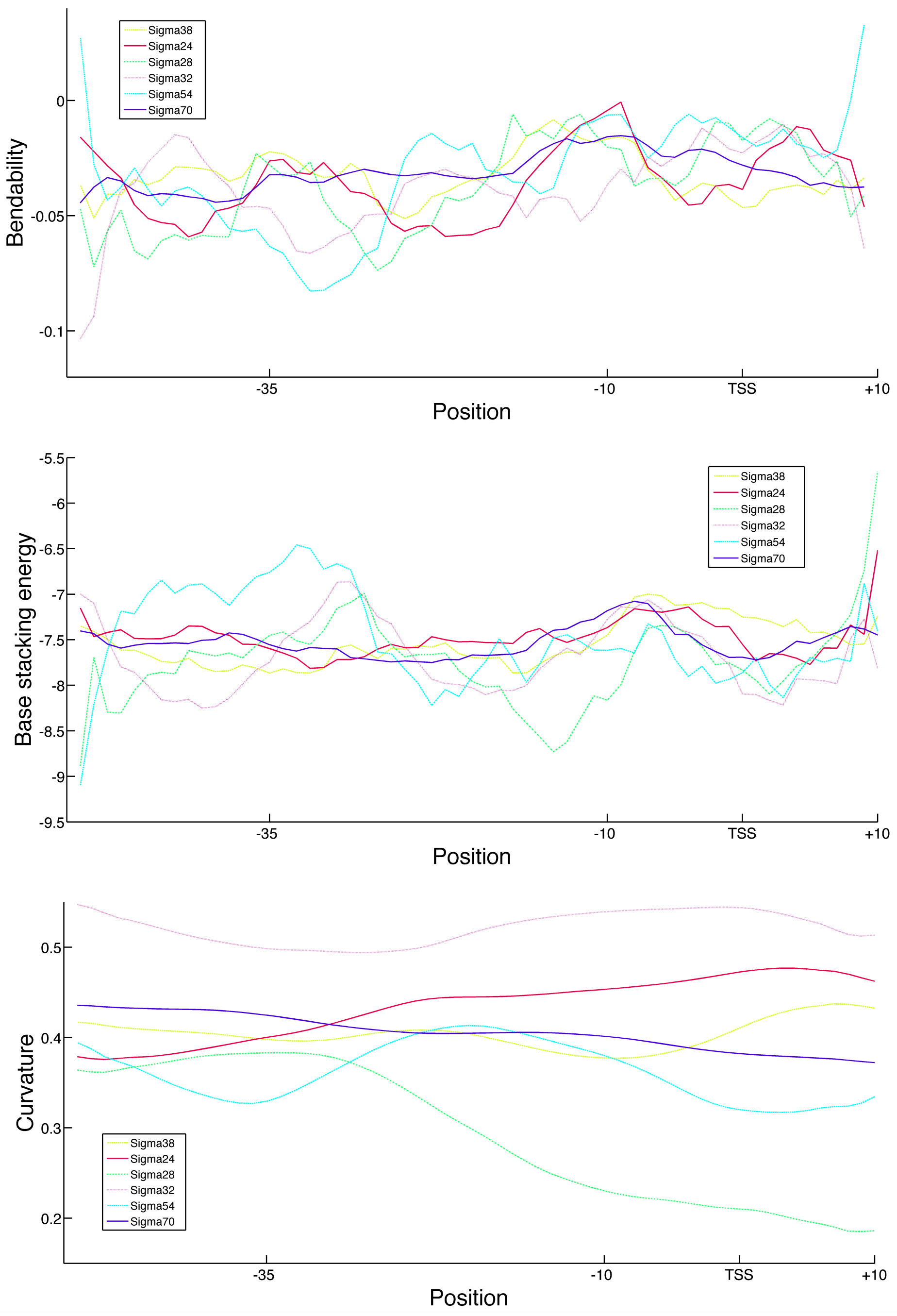

Supplemental figure S3: Average structural profiles for promoters annotated to be regulated by a given sigma factor smoothed with a span of 5. Each profile corresponds to a single sigma factor: Sigma 38 (light green dotted line), Sigma 24 (full red line), Sigma 28 (green dash-dotted line), Sigma 32 (dashed purple line), Sigma 54 (cyan dotted line) and Sigma 70 (full blue line). Sigma 19 is excluded as it only has one annotated promoter. Top: Average bendability profiles for promoters annotated to be regulated by the given sigma factor with high values corresponding to higher flexibility. Middle: Average base stacking profiles for promoters annotated to be regulated by the given sigma factor with higher values corresponding to lower stability. Bottom: Average curvature profiles for promoters annotated by the given sigma factor where higher values correspond to more curvature.
