# Supplementary material for: Structural Properties of Prokaryotic Promoter Regions Correlate with Functional Features
Source: PLoS One. 2014 Feb 7;9(2):e88717. doi: 10.1371/journal.pone.0088717 (PMC3918002; doi:10.1371/journal.pone.0088717)
Supplement: Figure S4 — Motif logos generated with WebLogo [50] of the promoter sequences from 50 bp upstream to 10 bp downstream from the TSS. Each set is grouped by the expression class of the first downstream gene, namely the stress expression class, general metabolism expression class, and growth expression class. (PDF) [file pone.0088717.s004.pdf]

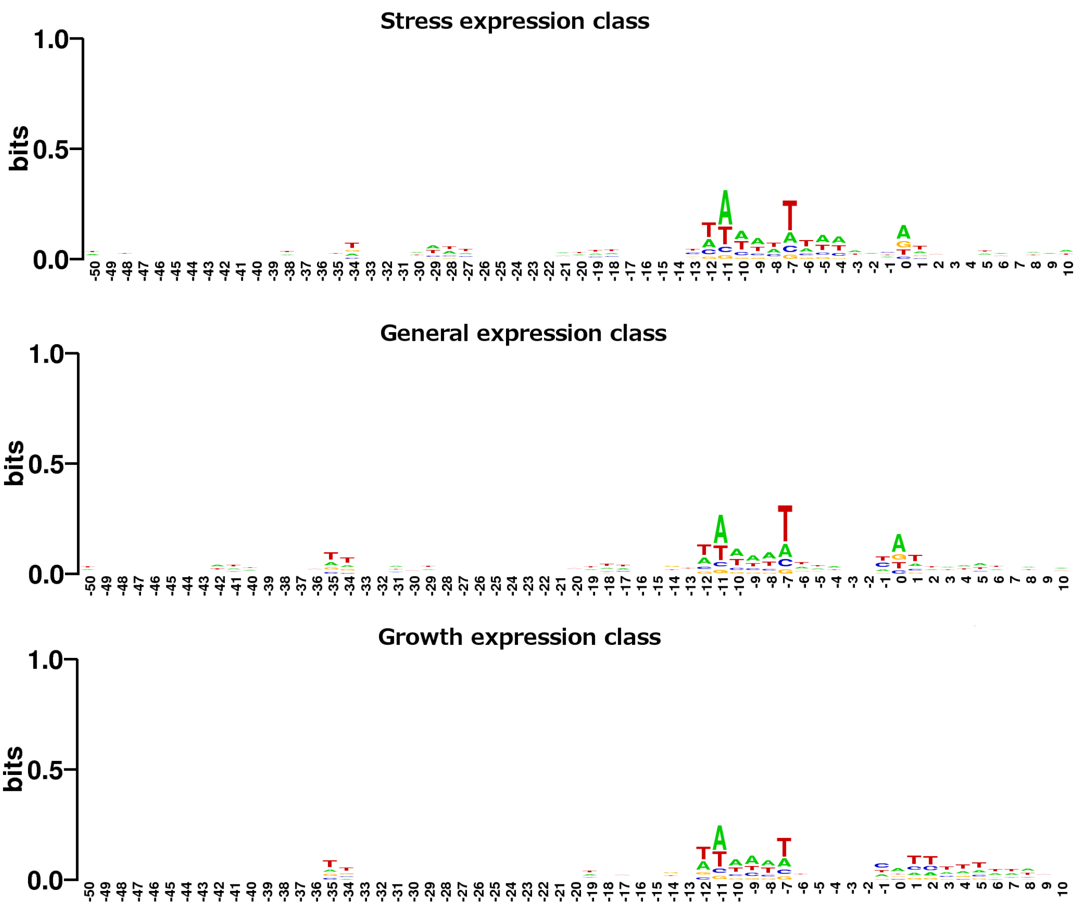

Supplemental figure S4: Motif logos generated with WebLogo of the promoter sequences from 50bp upstream to 10bp downstream from the TSS. Each set is grouped by the expression class of the first downstream gene, namely the stress expression class (top), general metabolism expression class (middle) and growth expression class (bottom). Note the y-axis only covers 1 bit instead of the standard 2 bits for motif logos, so that the very degenerate consensus sequences are still legible.
