# Supplementary material for: Structural Properties of Prokaryotic Promoter Regions Correlate with Functional Features
Source: PLoS One. 2014 Feb 7;9(2):e88717. doi: 10.1371/journal.pone.0088717 (PMC3918002; doi:10.1371/journal.pone.0088717)
Supplement: Figure S5 — Average base stacking profiles of the three expression classes grouped by experimental method used to determine the TSS. (PDF) [file pone.0088717.s005.pdf]

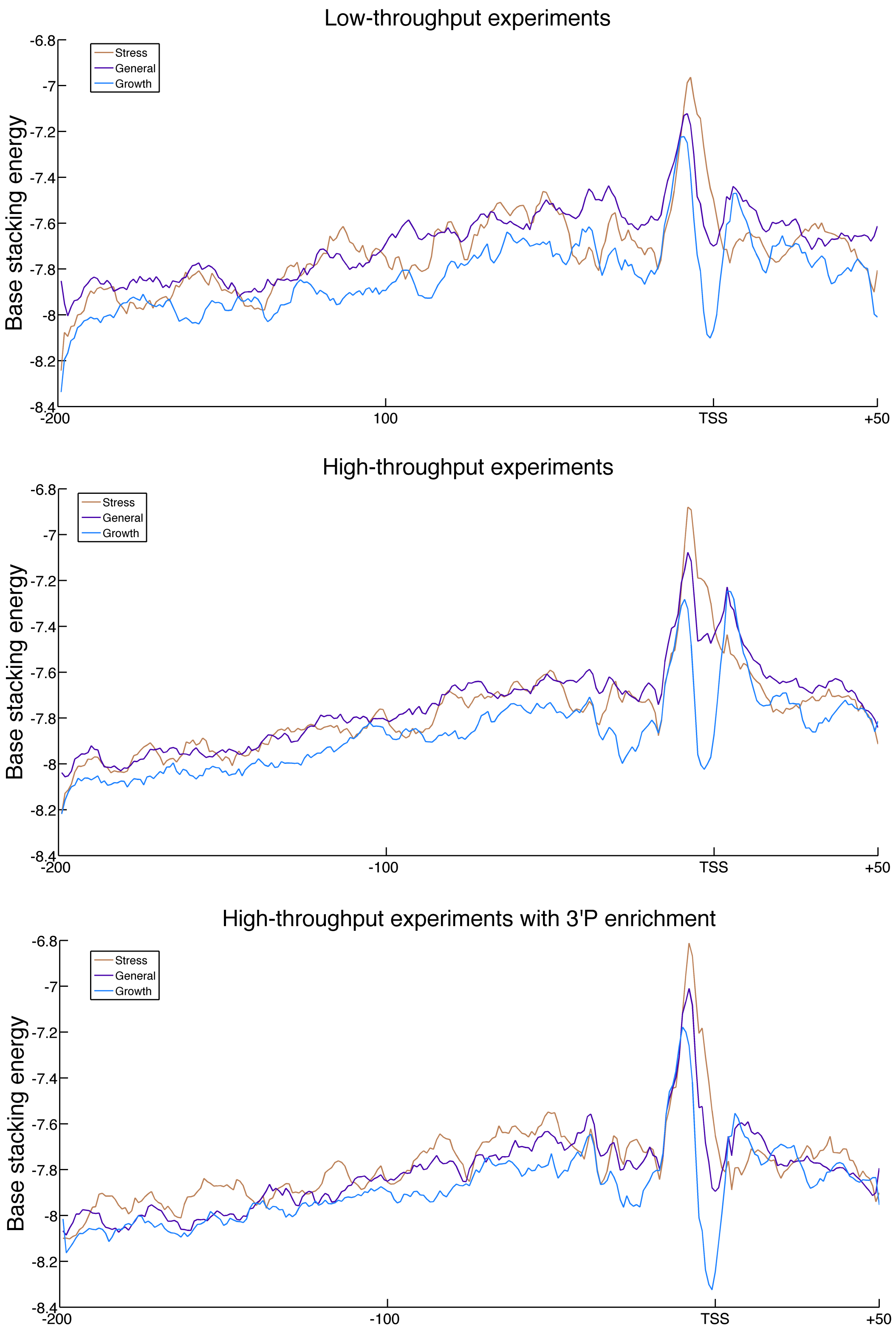

Supplemental figure 5: Average base stacking profiles of the three expression classes grouped by experimental method used to determine the TSS.
