# Supplementary material for: Structural Properties of Prokaryotic Promoter Regions Correlate with Functional Features
Source: PLoS One. 2014 Feb 7;9(2):e88717. doi: 10.1371/journal.pone.0088717 (PMC3918002; doi:10.1371/journal.pone.0088717)
Supplement: Figure S6 — Average base stacking energy profiles of the three experimental categories used to determine the TSS location grouped by functional expression class. (PDF) [file pone.0088717.s006.pdf]

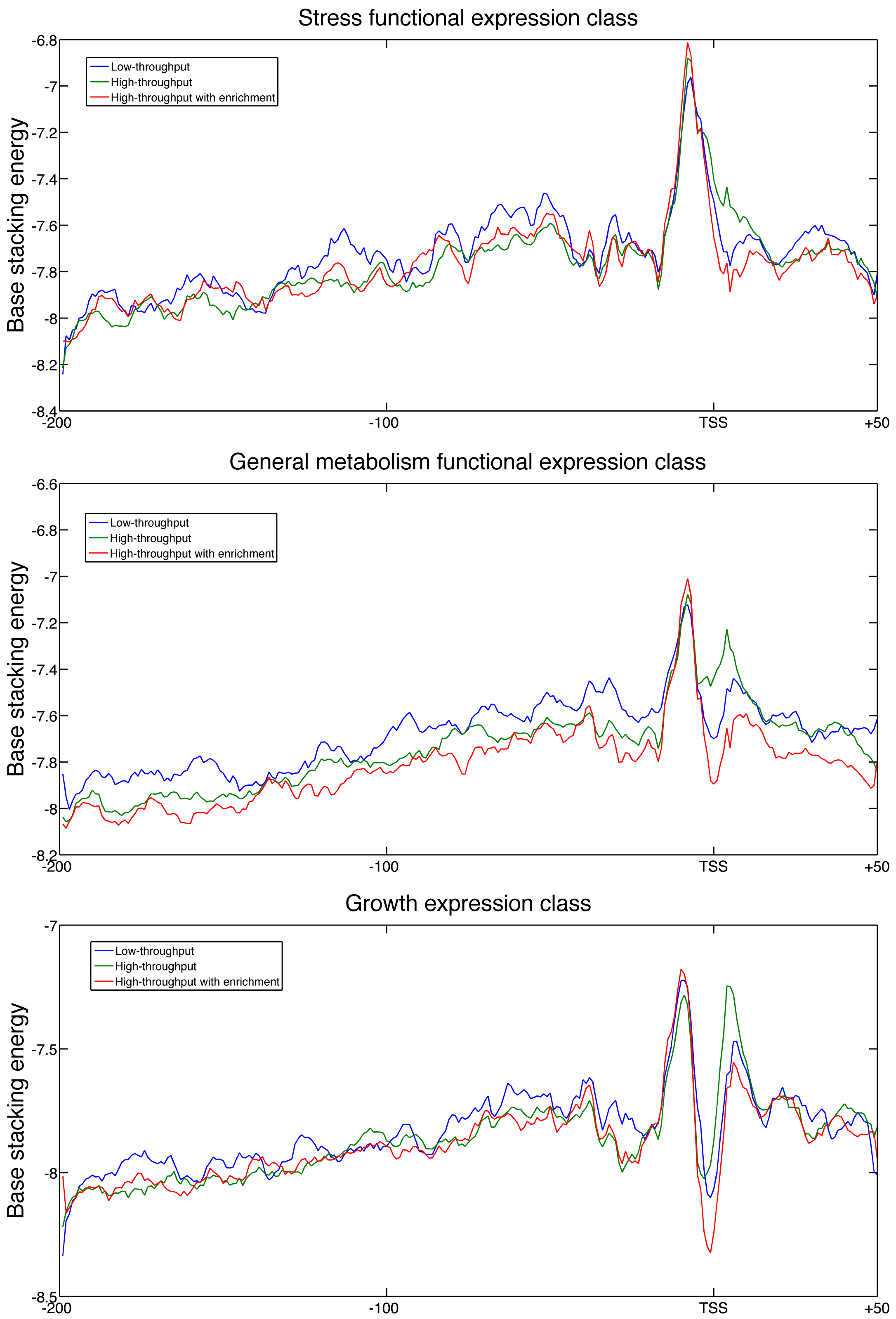

Supplemental figure S6: Average base stacking energy profiles of the three experimental categories used to determine the TSS location grouped by functional expression class.
