# Supplementary material for: Structural Properties of Prokaryotic Promoter Regions Correlate with Functional Features
Source: PLoS One. 2014 Feb 7;9(2):e88717. doi: 10.1371/journal.pone.0088717 (PMC3918002; doi:10.1371/journal.pone.0088717)
Supplement: Figure S8 — Motif logos generated with WebLogo [50] of the promoter regions (50 bp upstream to 10 bp downstream the TSS) from the six studied species from different phyla. (PDF) [file pone.0088717.s008.pdf]

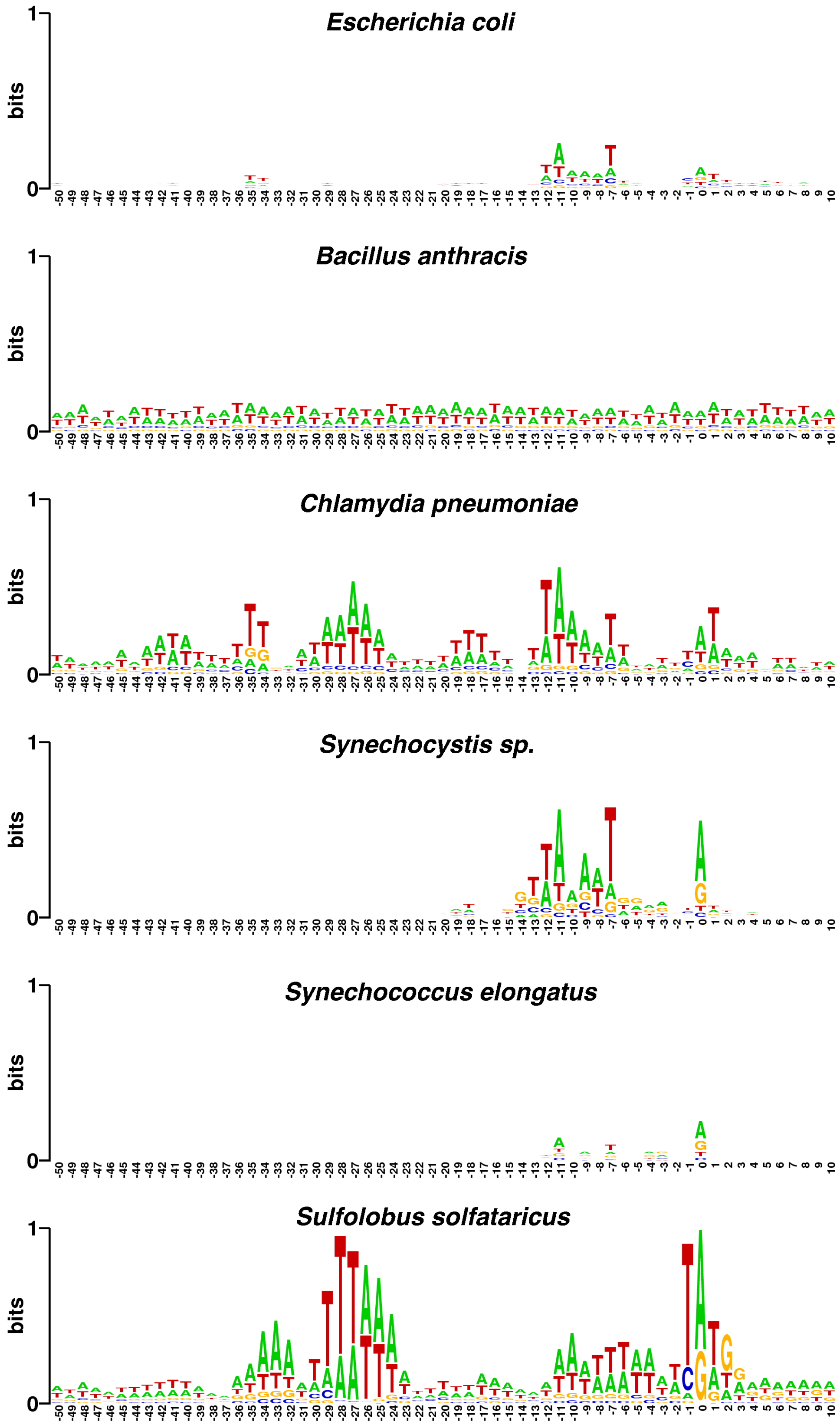

Supplemental figure S8: Motif logos generated with Weblogo of the promoter regions (50bp upstream to 10bp downstream of the TSS) for the six prokaryotes from different phyla.
