# Supplementary material for: Structural Properties of Prokaryotic Promoter Regions Correlate with Functional Features
Source: PLoS One. 2014 Feb 7;9(2):e88717. doi: 10.1371/journal.pone.0088717 (PMC3918002; doi:10.1371/journal.pone.0088717)
Supplement: Data S1 — Gene ontology enrichment results for each of the three expression classes compared against the set of all genes for which expression data is available. (PDF) [file pone.0088717.s009.pdf]

## Supplemental data S1: Gene ontology enrichment of the expression class genes

Here we report the ontology enrichments for each of the three expression classes compared against the set of all genes for which we have expression data. A more detailed analysis on the function of these classes can be found in Meysman *et al.* (2013).

### Gene ontology enrichments of the *E. coli* stress expression class genes

organic substance catabolic process : $2.11 \cdot 10^{-7}$ (110 of 394)
cellular catabolic process : $4.78 \cdot 10^{-8}$ (103 of 353)
oxidation-reduction process : $7.82 \cdot 10^{-8}$ (130 of 479)
carbohydrate catabolic process : $4.64 \cdot 10^{-7}$ (41 of 107)
lipid modification : $7.20538195616898 \cdot 10^{-5}$ (9 of 13)
response to heat : $6.05566537641835 \cdot 10^{-8}$ (20 of 33)
response to reactive oxygen species : $7.156 \cdot 10^{-5}$ (13 of 24)
carboxylic acid catabolic process : $3.533 \cdot 10^{-11}$ (57 of 136)
fatty acid catabolic process : $6.12 \cdot 10^{-6}$ (10 of 13)
organic acid catabolic process : $3.53 \cdot 10^{-11}$ (57 of 136)
cellular lipid catabolic process : $1.17 \cdot 10^{-5}$ (11 of 16)
response to oxidative stress : $1.01 \cdot 10^{-6}$ (25 of 53)
single-organism carbohydrate catabolic process : $2.47 \cdot 10^{-7}$ (38 of 94)
small molecule catabolic process : $2.19 \cdot 10^{-12}$ (69 of 171)
succinate metabolic process : $3.50 \cdot 10^{-5}$ (6 of 6)
response to stress : $1.08 \cdot 10^{-13}$ (89 of 236)
fatty acid beta-oxidation : $6.30 \cdot 10^{-6}$ (7 of 7)
catabolic process : $3.63 \cdot 10^{-8}$ (115 of 405)
monocarboxylic acid catabolic process : $3.95 \cdot 10^{-7}$ (29 of 64)
cellular carbohydrate catabolic process : $4.25 \cdot 10^{-6}$ (19 of 37)
lipid catabolic process : $1.91 \cdot 10^{-6}$ (13 of 19)
response to stimulus : $2.23 \cdot 10^{-6}$ (110 of 411)
single-organism catabolic process : $2.19 \cdot 10^{-12}$ (69 of 171)

### Gene ontology enrichments of the *E. coli* general metabolism expression class genes

transport : $8.40 \cdot 10^{-12}$ (435 of 694)
signal transduction : $2.74 \cdot 10^{-5}$ (72 of 101)
biological adhesion : $1.74 \cdot 10^{-9}$ (44 of 48)
cell adhesion : $5.94 \cdot 10^{-8}$ (38 of 42)
single-organism transport : $1.53 \cdot 10^{-7}$ (271 of 431)
anion transport : $2.84 \cdot 10^{-5}$ (47 of 61)
single-organism process : $1.12 \cdot 10^{-6}$ (460 of 783)
regulation of transcription, DNA-dependent : $2.80 \cdot 10^{-5}$ (224 of 365)
transmembrane transport : $3.52 \cdot 10^{-5}$ (160 of 252)

### Gene ontology enrichments of the *E. coli* growth expression class genes

cellular lipid metabolic process : $5.55 \cdot 10^{-8}$ (81 of 161)
RNA modification : $1.34 \cdot 10^{-11}$ (40 of 53)
pyridine-containing compound biosynthetic process : $1.75 \cdot 10^{-5}$ (15 of 19)
carbohydrate derivative biosynthetic process : $3.48 \cdot 10^{-16}$ (104 of 175)
alpha-amino acid biosynthetic process : $3.31 \cdot 10^{-8}$ (60 of 108)
ribose phosphate metabolic process : $4.04 \cdot 10^{-14}$ (37 of 43)
RNA processing : $2.28 \cdot 10^{-10}$ (43 of 62)

purine ribonucleoside monophosphate biosynthetic process : $4.98 \cdot 10^{-9}$ (20 of 22)
ribonucleoside monophosphate metabolic process : $1.74 \cdot 10^{-12}$ (32 of 37)
ribonucleotide metabolic process : $6.52 \cdot 10^{-8}$ (82 of 164)
heterocycle metabolic process : $7.44 \cdot 10^{-15}$ (215 of 465)
nucleobase-containing compound metabolic process : $4.27 \cdot 10^{-20}$ (240 of 495)
single-organism biosynthetic process : $1.42 \cdot 10^{-18}$ (140 of 249)
oligosaccharide biosynthetic process : $5.03 \cdot 10^{-6}$ (28 of 44)
purine nucleotide metabolic process : $1.24 \cdot 10^{-5}$ (71 of 152)
macromolecule modification : $9.56 \cdot 10^{-6}$ (66 of 138)
phosphate-containing compound metabolic process : $6.67 \cdot 10^{-6}$ (203 of 524)
cellular amino acid metabolic process : $1.10 \cdot 10^{-8}$ (128 of 279)
lipid metabolic process : $2.52 \cdot 10^{-7}$ (82 of 168)
ncRNA processing : $2.01 \cdot 10^{-10}$ (36 of 48)
tRNA aminoacylation : $1.08 \cdot 10^{-9}$ (23 of 26)
intracellular protein transport : $1.87 \cdot 10^{-7}$ (13 of 13)
Gram-negative-bacterium-type cell outer membrane assembly : $6.75 \cdot 10^{-6}$ (10 of 10)
pteridine-containing compound metabolic process : $5.04 \cdot 10^{-5}$ (15 of 20)
hydrogen transport : $2.41 \cdot 10^{-7}$ (24 of 32)
cellular biosynthetic process : $5.59 \cdot 10^{-30}$ (284 of 552)
cellular macromolecule biosynthetic process : $8.96 \cdot 10^{-6}$ (54 of 107)
cellular nitrogen compound biosynthetic process : $1.06 \cdot 10^{-22}$ (157 of 271)
purine ribonucleotide biosynthetic process : $5.68 \cdot 10^{-11}$ (24 of 26)
glycosyl compound biosynthetic process : $9.49 \cdot 10^{-6}$ (28 of 45)
ncRNA metabolic process : $1.29 \cdot 10^{-17}$ (59 of 76)
nucleoside biosynthetic process : $9.49 \cdot 10^{-6}$ (28 of 45)
sulfur compound biosynthetic process : $6.01 \cdot 10^{-6}$ (35 of 60)
ribose phosphate biosynthetic process : $4.04 \cdot 10^{-14}$ (37 of 43)
macromolecule metabolic process : $1.11 \cdot 10^{-18}$ (212 of 430)
heterocycle biosynthetic process : $1.97 \cdot 10^{-19}$ (145 of 257)
tetrahydrofolate metabolic process : $1.02 \cdot 10^{-5}$ (13 of 15)
lipopolysaccharide core region biosynthetic process : $2.73 \cdot 10^{-8}$ (20 of 23)
organophosphate biosynthetic process : $7.52 \cdot 10^{-16}$ (112 of 195)
purine nucleoside monophosphate metabolic process : $4.98 \cdot 10^{-9}$ (20 of 22)
O antigen biosynthetic process : $2.23 \cdot 10^{-5}$ (9 of 9)
thiamine diphosphate metabolic process : $5.37 \cdot 10^{-5}$ (10 of 11)
pyrimidine ribonucleotide metabolic process : $2.92 \cdot 10^{-5}$ (12 of 14)
membrane assembly : $6.75 \cdot 10^{-6}$ (10 of 10)
nucleobase-containing compound biosynthetic process : $4.08 \cdot 10^{-14}$ (92 of 156)
alpha-amino acid metabolic process : $4.13 \cdot 10^{-5}$ (83 of 189)
phosphorus metabolic process : $1.91 \cdot 10^{-5}$ (206 of 540)
membrane organization : $2.04 \cdot 10^{-6}$ (11 of 11)
monovalent inorganic cation transport : $6.30 \cdot 10^{-7}$ (24 of 33)
vitamin biosynthetic process : $5.11 \cdot 10^{-10}$ (43 of 63)
nucleotide metabolic process : $1.07 \cdot 10^{-10}$ (127 of 261)
translation : $6.59 \cdot 10^{-43}$ (95 of 101)
fatty acid biosynthetic process : $2.94 \cdot 10^{-6}$ (18 of 23)
cofactor metabolic process : $5.64 \cdot 10^{-11}$ (71 of 121)
purine-containing compound biosynthetic process : $2.65 \cdot 10^{-7}$ (27 of 38)
lipopolysaccharide core region metabolic process : $2.73 \cdot 10^{-8}$ (20 of 23)
carbohydrate biosynthetic process : $7.43 \cdot 10^{-6}$ (64 of 132)
cellular aromatic compound metabolic process : $1.01 \cdot 10^{-9}$ (187 of 432)
polysaccharide biosynthetic process : $2.23 \cdot 10^{-5}$ (51 of 102)
tRNA modification : $1.60 \cdot 10^{-10}$ (34 of 44)
purine nucleoside monophosphate biosynthetic process : $4.98 \cdot 10^{-9}$ (20 of 22)
lipopolysaccharide biosynthetic process : $4.11 \cdot 10^{-6}$ (43 of 78)
purine ribonucleotide metabolic process : $1.78 \cdot 10^{-5}$ (69 of 148)
pteridine-containing compound biosynthetic process : $2.92 \cdot 10^{-5}$ (12 of 14)
energy coupled proton transmembrane transport, against electrochemical gradient : $2.23 \cdot 10^{-5}$ (9 of 9)

lipid biosynthetic process : $8.61 \cdot 10^{-10}$ (70 of 124)
guanosine-containing compound metabolic process : $1.50\cdot 10^{-6}$ (22 of 30)
coenzyme biosynthetic process : $1.10 \cdot 10^{-11}$ (55 of 83)
tRNA aminoacylation for protein translation : $3.19 \cdot 10^{-9}$ (22 of 25)
tRNA methylation : $6.75 \cdot 10^{-6}$ (10 of 10)
tRNA processing : $5.07 \cdot 10^{-10}$ (35 of 47)
ATP synthesis coupled proton transport : $2.23 \cdot 10^{-5}$ (9 of 9)
cellular macromolecule metabolic process : $2.78 \cdot 10^{-20}$ (185 of 351)
rRNA modification : $3.69 \cdot 10^{-6}$ (22 of 31)
pyrimidine nucleotide biosynthetic process : $2.44 \cdot 10^{-6}$ (17 of 21)
pyrimidine ribonucleotide biosynthetic process : $2.93 \cdot 10^{-5}$ (12 of 14)
water-soluble vitamin metabolic process : $9.88 \cdot 10^{-8}$ (44 of 73)
nucleoside phosphate biosynthetic process : $8.11 \cdot 10^{-15}$ (71 of 107)
protein peptidyl-prolyl isomerization : $5.38 \cdot 10^{-5}$ (10 of 11)
organic substance biosynthetic process : $5.20 \cdot 10^{-32}$ (282 of 536)
'de novo' IMP biosynthetic process : $6.18 \cdot 10^{-7}$ (12 of 12)
macromolecule methylation : $1.50 \cdot 10^{-11}$ (34 of 42)
ribonucleotide biosynthetic process : $4.04 \cdot 10^{-15}$ (36 of 40)
small molecule biosynthetic process : $2.82 \cdot 10^{-18}$ (139 of 248)
organophosphate metabolic process : $6.51 \cdot 10^{-12}$ (170 of 366)
cofactor biosynthetic process : $4.71 \cdot 10^{-9}$ (60 of 104)
pyrimidine ribonucleoside biosynthetic process : $2.93 \cdot 10^{-5}$ (12 of 14)
organic acid biosynthetic process : $1.32 \cdot 10^{-11}$ (100 of 187)
biosynthetic process : $1.17 \cdot 10^{-29}$ (300 of 596)
ribonucleoside monophosphate biosynthetic process : $9.89 \cdot 10^{-13}$ (31 of 35)
methylation : $3.98 \cdot 10^{-11}$ (46 of 66)
O antigen metabolic process : $2.23 \cdot 10^{-5}$ (9 of 9)
water-soluble vitamin biosynthetic process : $5.11 \cdot 10^{-10}$ (43 of 63)
rRNA base methylation : $1.75 \cdot 10^{-5}$ (15 of 19)
rRNA methylation : $2.11 \cdot 10^{-5}$ (17 of 23)
energy coupled proton transport, down electrochemical gradient : $2.22 \cdot 10^{-5}$ (9 of 9)
cellular polysaccharide biosynthetic process : $2.68 \cdot 10^{-5}$ (50 of 100)
lipopolysaccharide metabolic process : $6.42 \cdot 10^{-6}$ (43 of 79)
carboxylic acid biosynthetic process : $1.32 \cdot 10^{-11}$ (100 of 187)
RNA methylation : $1.72 \cdot 10^{-9}$ (28 of 35)
nucleotide biosynthetic process : $8.11 \cdot 10^{-15}$ (71 of 107)
organonitrogen compound biosynthetic process : $6.66 \cdot 10^{-22}$ (173 of 313)
organic substance metabolic process : $1.87 \cdot 10^{-5}$ (405 of 1156)
pyrimidine-containing compound biosynthetic process : $1.10 \cdot 10^{-7}$ (28 of 39)
cellular nitrogen compound metabolic process : $1.39 \cdot 10^{-13}$ (239 of 542)
macromolecule biosynthetic process : $4.79 \cdot 10^{-8}$ (76 of 148)
nucleoside monophosphate biosynthetic process : $2.77 \cdot 10^{-12}$ (33 of 39)
proton transport : $2.41 \cdot 10^{-7}$ (24 of 32)
tRNA metabolic process : $3.43 \cdot 10^{-17}$ (58 of 75)
rRNA processing : $4.09 \cdot 10^{-8}$ (27 of 36)
small molecule metabolic process : $2.39 \cdot 10^{-5}$ (297 of 819)
coenzyme metabolic process : $5.39 \cdot 10^{-9}$ (65 of 116)
purine nucleotide biosynthetic process : $4.08 \cdot 10^{-11}$ (26 of 29)
organic cyclic compound biosynthetic process : $1.19 \cdot 10^{-19}$ (145 of 256)
thiamine diphosphate biosynthetic process : $5.38 \cdot 10^{-5}$ (10 of 11)
aromatic compound biosynthetic process : $1.03 \cdot 10^{-18}$ (125 of 213)
carbohydrate derivative metabolic process : $4.91 \cdot 10^{-11}$ (160 of 346)
cellular amino acid biosynthetic process : $1.53 \cdot 10^{-8}$ (82 of 160)
cellular membrane organization : $2.04 \cdot 10^{-6}$ (11 of 11)
folic acid-containing compound biosynthetic process : $2.92 \cdot 10^{-5}$ (12 of 14)
ribonucleoside biosynthetic process : $2.57 \cdot 10^{-6}$ (28 of 43)
pyrimidine nucleotide metabolic process : $2.44 \cdot 10^{-6}$ (17 of 21)
amino acid activation : $5.20 \cdot 10^{-9}$ (23 of 27)
nucleoside monophosphate metabolic process : $4.03 \cdot 10^{-12}$ (34 of 41)

organonitrogen compound metabolic process : $1.36 \cdot 10^{-11}$ (244 of 575)
purine ribonucleoside monophosphate metabolic process : $4.98 \cdot 10^{-9}$ (20 of 22)
IMP metabolic process : $1.70 \cdot 10^{-8}$ (15 of 15)
nucleoside phosphate metabolic process : $1.48 \cdot 10^{-10}$ (127 of 262)
organic cyclic compound metabolic process : $4.88 \cdot 10^{-11}$ (215 of 498)
RNA metabolic process : $7.11 \cdot 10^{-17}$ (83 of 126)
dicarboxylic acid biosynthetic process : $6.15 \cdot 10^{-7}$ (27 of 39)
nucleic acid metabolic process : $3.04 \cdot 10^{-15}$ (101 of 172)
IMP biosynthetic process : $1.70 \cdot 10^{-8}$ (15 of 15)
vitamin metabolic process : $9.88 \cdot 10^{-8}$ (44 of 73)
nucleobase-containing small molecule metabolic process : $1.73 \cdot 10^{-7}$ (139 of 320)
nitrogen compound metabolic process : $1.05 \cdot 10^{-11}$ (295 of 722)
peptidyl-proline modification : $5.38 \cdot 10^{-5}$ (10 of 11)
